# Application of metal-free conditions to a one-pot Leimgruber–Batcho indole synthesis

**DOI:** 10.1039/d6ra02702d

**Published:** 2026-05-19

**Authors:** Beate Halsvik, Bengt Erik Haug

**Affiliations:** a University of Bergen, Department of Chemistry and Centre for Pharmacy Allégaten 41 5020 Bergen Norway bengt-erik.haug@uib.no

## Abstract

A metal-free, one-pot synthesis of 2,3-unsubstituted indoles *via* an adapted Leimgruber–Batcho protocol has been developed. The process incorporates a chemoselective reduction of an aromatic nitro group using tetrahydroxydiboron and 4,4′-bipyridine, enabling efficient access to indole scaffolds under mild conditions. Application of this method resulted in comparable or improved yields relative to existing metal-based protocols. The successful preparation of 6-bromo-5-methoxyindole, a key constituent of breitfussin C, G, and H, shows the utility of this method for the synthesis of indoles relevant to natural product synthesis.

## Abstract 2

A metal-free, one-pot synthesis of 2,3-unsubstituted indoles *via* an adapted Leimgruber–Batcho protocol has been developed.

## Graphical abstract

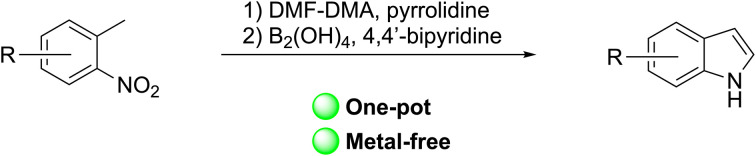

## Introduction

Indoles are structurally versatile and pharmacologically privileged heterocycles found in a wide array of biologically active natural products, pharmaceuticals, and agrochemicals.^1–3^ Notably, indole motifs are found in marine natural products such as the breitfussins, which exhibit promising cytotoxic properties.^4–6^ Among the various substitution patterns, 2,3-unsubstituted indoles are valuable due to their utility as synthetic intermediates in medicinal chemistry and materials science. This significance has been thoroughly documented in both foundational^7–9^ and recent^10–12^ studies.

**Fig. 1:**
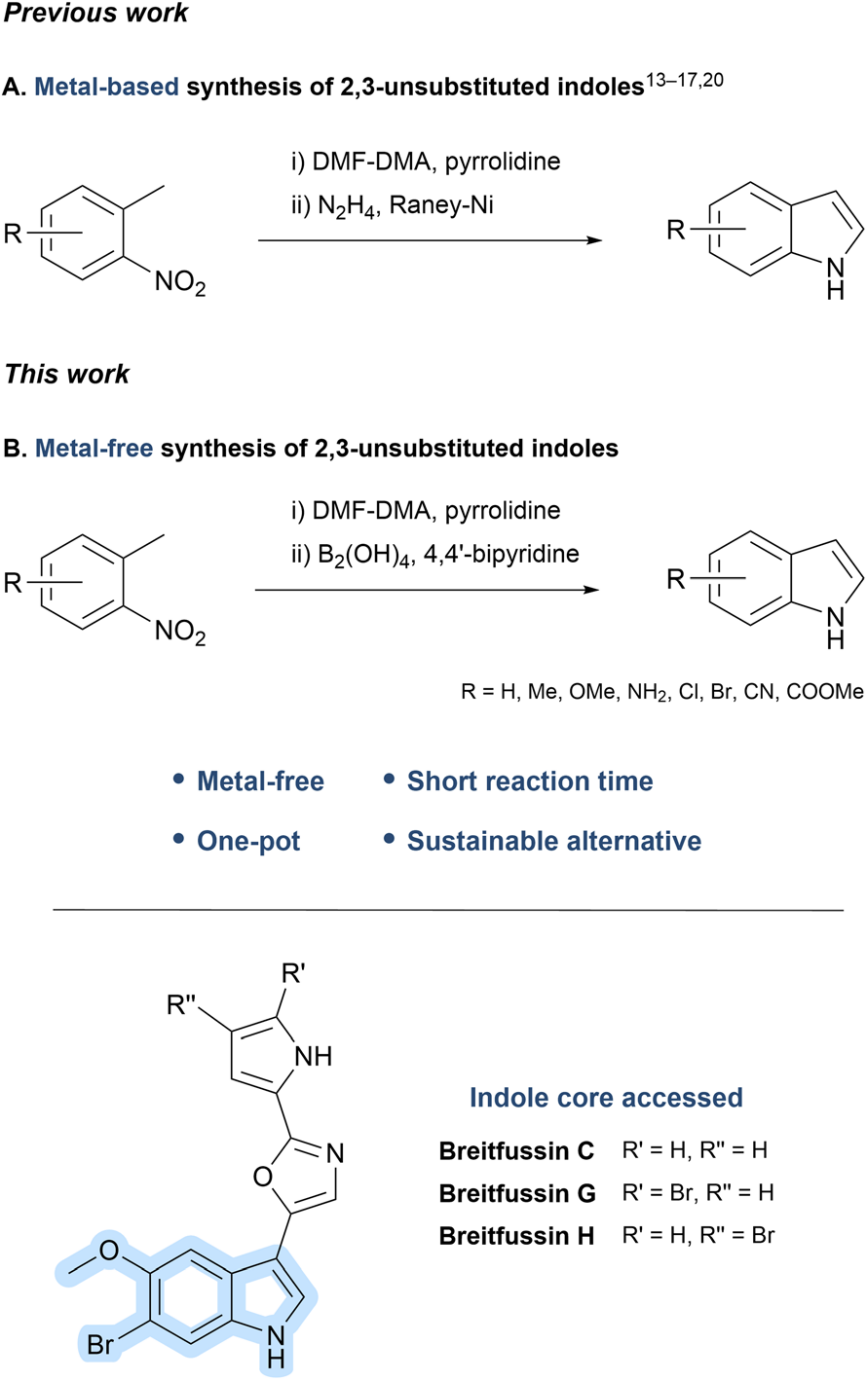
Classical, tandem, and metal-free one-pot syntheses of 2,3-unsubstituted indoles with application to breitfussin C, G and H.

Among the synthetic strategies available for constructing 2,3-unsubstituted indoles, the classical Leimgruber–Batcho reaction (Fig. 1) stands out for its functional group tolerance and scalability.^13–17^ This two-step process involves converting the methyl group of *o*-nitrotoluenes into an enamine using DMF-DMA/pyrrolidine, followed by reductive cyclisation of the enamine with Raney nickel and hydrazine.^18^ However, this traditional approach often entails prolonged reaction times and challenging isolation of intermediates.

To address these limitations, numerous modifications of the Leimgruber–Batcho synthesis have been reported.^17,19,20^ Among these, Chen *et al.* reported a microwave-assisted tandem one-pot synthesis of 2,3-unsubstituted indoles, delivering higher yields and shorter reaction times compared to the classical stepwise approach.^20^ While effective, the use of transition metals and hydrazine raises concerns regarding environmental impact and safety.

Jang *et al.* recently reported a metal-free, chemoselective reduction of aromatic nitro compounds using tetrahydroxydiboron [B_2_(OH)_4_] and 4,4′-bipyridine.^21^ Among the substrates they examined, the reduction of an *o*-allyl nitrobenzene to 2-allylaniline, with the double bond fully preserved, was particularly relevant to our study, as it suggested that the enamine intermediate of the Leimgruber–Batcho reaction might likewise tolerate these conditions. Błyszczyk and Roure have also recently demonstrated the photochemical use of tetrahydroxydiboron in nitroarene reductions, further highlighting the reagent's versatility in more sustainable transformations.^22^ Motivated by these observations, we sought to investigate whether a metal-free approach could be integrated into the Leimgruber–Batcho indole synthesis.

In this work, we evaluated a series of twelve structurally and electronically diverse *o*-nitrotoluenes using a metal-free reduction strategy (Fig. 1) inspired by the work of Jang *et al.*^21^ Integrating this chemoselective reduction into a streamlined, metal-free, one-pot adaptation of the Leimgruber–Batcho indole synthesis enabled more sustainable, efficient access to 2,3-unsubstituted indoles under mild conditions.

Across this broad substrate set, we achieved yields comparable to or exceeding those of established metal-based protocols. Notably, our approach was applied to the preparation of 6-bromo-5-methoxyindole, the core structural motif of the marine natural products breitfussin C, G, and H, thereby enabling the efficient synthesis of this key intermediate and offering a promising alternative for future synthetic efforts toward halogenated indole-containing marine natural products.

## Results and discussion

### Initial validation of a metal-free one-pot indole synthesis

The idea for this metal-free one-pot synthesis was inspired by the reported chemoselective reduction of aromatic nitro groups by Jang *et al.*,^21^ which showed the clean conversion of aromatic nitro compounds to anilines using tetrahydroxydiboron and 4,4′-bipyridine in DMF. We recognised that this mild bipyridine-catalysed reduction might be compatible with the key enamine intermediate of the Leimgruber–Batcho indole synthesis. In this transformation, the nitro group is selectively reduced to trigger cyclisation following the condensation step. This raised the possibility of integrating the two steps into a streamlined, metal-free one-pot protocol. We found it significant that both the Leimgruber–Batcho condensation and the bipyridine-catalysed reduction are optimally performed in DMF.^15,21^

**Scheme 1:**
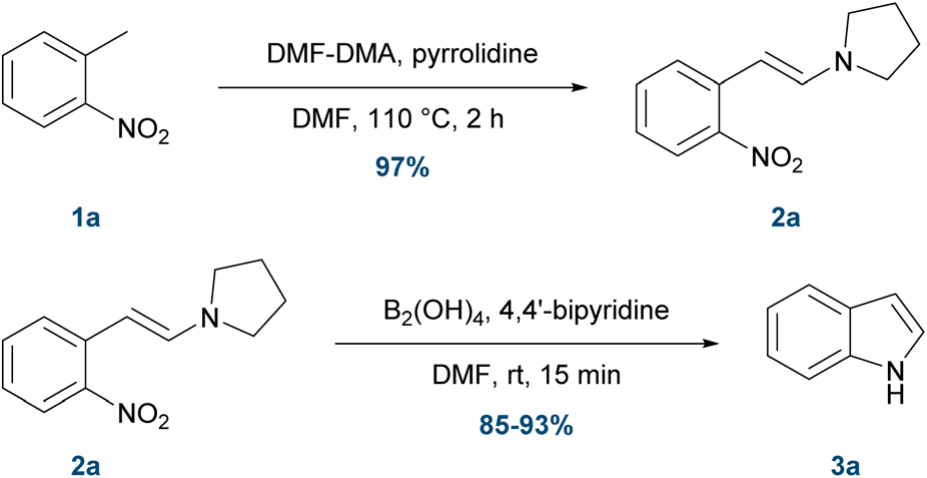
Stepwise, small-scale validation of a metal-free indole synthesis.

To establish proof of concept, we first replicated the initial condensation step of the classical Leimgruber–Batcho synthesis using *o*-nitrotoluene (1a) as a model substrate. Following the original procedure,^15^1a (7.0 mmol) was treated with 1.2 equivalents of DMF-DMA and pyrrolidine in DMF at 110 °C for 2 h, affording the enamine intermediate (*E*)-1-(2-nitrostyryl)-pyrrolidine (2a) in 97% isolated yield (Scheme 1). The isolated enamine was then subjected to the metal-free nitro reduction conditions reported by Jang *et al.*,^21^ using 4.0 equivalents of tetrahydroxydiboron and 0.5 mol% 4,4′-bipyridine in DMF. The corresponding indole 3a was obtained in 85–93% isolated yield from two parallel reactions on a 0.2 mmol scale.

**Scheme 2:**
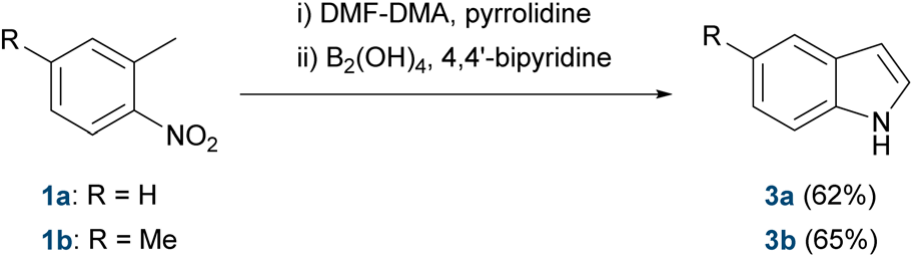
Small-scale validation of a metal-free one-pot indole synthesis.

We next tested the full one-pot sequence (Scheme 2), where 1a (0.7 mmol) was treated with DMF-DMA and pyrrolidine under the same conditions as above. After 2 h at reflux, the reaction mixture was cooled to room temperature, and tetrahydroxydiboron and 4,4′-bipyridine were added directly. After 15 minutes of stirring, TLC analysis indicated complete conversion. The work-up and purification afforded the desired 2,3-unsubstituted indole 3a in 62% isolated yield. A second substrate, 2,4-dimethyl-1-nitrobenzene (1b), gave the corresponding indole 3b under identical conditions in 65% isolated yield.

These initial results confirmed that the metal-free reduction conditions could be successfully integrated into a Leimgruber–Batcho-type annulation, enabling a concise, one-pot synthesis of indoles without transition metals or hazardous reductants.

### Refinement and scale-up of the one-pot protocol

Building upon the successful proof-of-concept experiments, the one-pot protocol was scaled to 4.0 mmol, as was used by Chen *et al.*^20^ A systematic exploration of key reaction parameters was undertaken to refine the methodology, with the outcomes summarised in Table 1.

**Table 1:** Refinement of the metal-free one-pot synthesis^a^

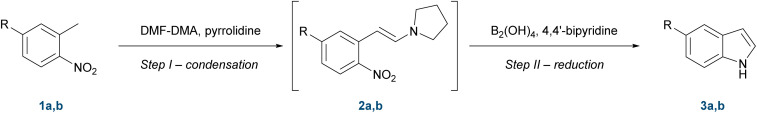								
Entry	R	Step I		Step II				Yield 3 (%)
		DMF-DMA (equiv.)	Pyrrolidine (equiv.)	B_2_(OH)_4_ (equiv.)	4,4′-Bipyridine (mol%)	Temp. (°C)	Time (min)	
1	H	1.5	1.5	4.0	0.5	21	30	28^b^
2	H	1.5	1.5	4.0	0.5	21	30	50
3	H	1.5	1.5	4.0	0.5	45	30	62
4	H	1.5	1.5	2 × 4.0	2 × 1.0	45	60	70
5	H	1.5	1.5	2 × 4.0	2 × 1.0	45	60	25^c^
6	H	2.0	2.0	2 × 4.0	2 × 1.0	45	60	77
7	H	2.5	2.5	2 × 4.0	2 × 1.0	45	60	65
8	H	3.0	3.0	2 × 4.0	2 × 1.0	45	60	58
9	Me	1.5	1.5	2 × 4.0	2 × 1.0	45	60	37
10	Me	1.5	1.5	2 × 4.0	2 × 1.0	45	60	7^c^
11	Me	2.0	2.0	2 × 4.0	2 × 1.0	45	60	44
12	Me	2.5	2.5	2 × 4.0	2 × 1.0	45	60	14
13	Me	3.0	3.0	2 × 4.0	2 × 1.0	45	60	11

a Reaction conditions: Step I – 1a or 1b (4.0 mmol), DMF (5 mL) at 110 °C for 2 h; Step II – 20 mL of DMF in total used as solvent.

b 20 mL of DMF used in Step I.

c Reaction time for Step I increased to 24 h.

Using 1a as a model substrate, we found that the condensation step did not go to completion when performing the reaction in 20 mL DMF, which resulted in a markedly lower yield of indole 3a (entry 1, 28%) than when the condensation was carried out in 5 mL DMF and diluted only prior to reduction (entry 2, 50%). Increasing the reduction temperature to 45 °C further improved the yield (entry 3), and combining elevated temperature with a two-portion addition of tetrahydroxydiboron and 4,4′-bipyridine yielded 3a in 70% (entry 4). This effective combination of heating and stepwise addition of reductant and catalyst was applied in all subsequent experiments.

Adjustments to the condensation step indicated that both extended reaction times and elevated reagent concentrations reduced reaction efficiency. Prolonging the reaction to 24 h led to decomposition of intermediate 2a, resulting in a challenging work-up and a significantly reduced yield (entry 5, 25%). Increasing the loading of DMF-DMA and pyrrolidine to 2.0 equivalents afforded the highest yield obtained for 1a (entry 6, 77%). A further increase to 2.5 equivalents resulted in a moderate reduction in yield (entry 7, 65%), and raising the loading to 3.0 equivalents (entry 8) led to a further decline. These results indicate that while a slight excess of reagents can support conversion, elevated stoichiometry also accelerates competing degradation pathways.

A comparable set of experiments was conducted using 2,4-dimethyl-1-nitrobenzene (1b). As anticipated, based on the proposed mechanism and prior literature on the Leimgruber–Batcho reaction,^15,20^ the overall reactivity was lower than that of the unsubstituted analogue 1a. It is worth noting that the trends were consistent. At 1.5 equivalents, yields were modest (entry 9, 37%), and extending the condensation time to 24 h (entry 10) or increasing the reagent loading to 3.0 equivalents (entry 13) resulted in substantially lower yields. Increasing the loading to 2.0 equivalents gave the highest yield (entry 11, 44%), whereas 2.5 equivalents led to a pronounced reduction in yield (entry 12, 14%).

These results collectively emphasise the sensitivity of the condensation step to both reagent stoichiometry and reaction concentration, stressing the need to balance conversion with stability of the intermediate. The reduction conditions, once established, yielded consistent, reproducible results. Based on these findings, a refined protocol was adopted for the subsequent substrate scope investigations. This included using 2.0 equivalents of DMF-DMA and pyrrolidine, heating at 110 °C for 2 h, cooling to 45 °C, followed by the addition of tetrahydroxydiboron (2 × 4.0 equivalents) and 4,4′-dipyridine (2 × 1.0 mol%), and stirring at 45 °C for 1 h.

Recent studies have shown that B_2_(OH)_4_-mediated nitro reductions can exhibit notable exothermicity under concentrated conditions.^23^ Consistent with these observations, dilute reaction conditions, combined with staged addition of tetrahydroxydiboron, afforded controlled and reproducible behaviour in the present one-pot system. In this context, Revu *et al.* reported a safer continuous-flow protocol for B_2_(OH)_4_-mediated chemoselective nitro reduction,^23^ suggesting that the present methodology could be compatible with future flow-based applications.

### Substrate scope of the metal-free one-pot protocol

We next evaluated the substrate scope by including twelve substituted *o*-nitrotoluenes (Table 2). This substrate scope was assembled to provide a broad representation of electronic and steric variation, incorporating substrates previously examined under the classical Leimgruber–Batcho conditions and in the one-pot procedure reported by Chen *et al.*,^20^ alongside additional derivatives selected to extend the structural diversity of the study. This combination enabled both benchmarking against existing methods and assessment of the wider applicability of the metal-free one-pot protocol. To further substantiate the synthetic utility, we applied it to the preparation of 6-bromo-5-methoxyindole (entry 12, 3l), a highly functionalised indole scaffold relevant to marine natural product synthesis.^4–6,24^

**Table 2:** Metal-free one-pot synthesis of indoles 3^a^

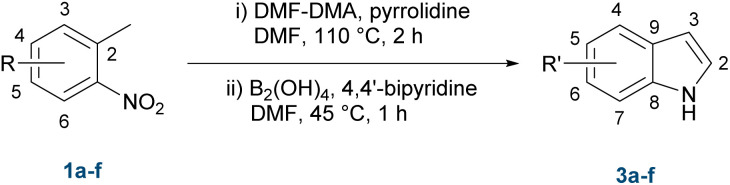						
Entry	R	1	R′	3	Lit. Y (%)	Y (%)^b^
1	H	1a	H	3a	71^c^, 80^d^	71–77
2	4-Me	1b	5-Me	3b	65^c^	41–44
3	3-NH_2_	1c	4-NH_2_	3c	34^c^	43–45
4	3-Cl	1d	4-Cl	3d	55^c^, 63^d^	66–67
5	4-Cl	1e	5-Cl	3e	74^c^, 78^d^	81–82
6	4-Br	1f	5-Br	3f	n.d.	64–68^e^
7	4-CN	1g	5-CN	3g	71^c^	74–76
8	4-COOMe	1h	5-COOMe	3h	n.d.	71–74
9	5-Br	1i	6-Br	3i	82^c^, 37^d^	53–58^e^
10	6-Me	1j	7-Me	3j	53^c^, 48^d^	40–42
11	4,5-Br	1k	5,6-Br	3k	n.d.	32–34^e^
12	5-Br,4-OMe	1l	6-Br, 5-OMe	3l	n.d.	58–62

a Reaction conditions: Step i – 1 (4.0 mmol), DMF-DMA (2.0 equiv.), pyrrolidine (2.0 equiv.), DMF (5 mL) at 110 °C for 2 h; Step ii – tetrahydroxydiboron (2 × 4.0 equiv.), 4,4′-bipyridine (2 × 1.0 mol%), DMF (20 mL) at 45 °C for 1 h.

b Isolated yields across two independent runs.

c Results reported by Chen *et al*.^20^

d Results reported in the original Leimgruber–Batcho publication.^15^

e Partial debromination observed. Yield estimated by ^1^H NMR.

Across the substrate set, clear electronic and steric trends emerged. The parent substrate 1a performed reliably under the optimised conditions and serves as a useful point of reference for assessing substituent effects. Electron-donating derivatives showed the anticipated decrease in efficiency: the 4-methyl substrate 1b afforded a noticeably lower yield, the strongly donating 3-amino substituent of 1c was moderately well tolerated, and the sterically hindered 6-methyl analogue 1j gave a similar yield.

Electron-withdrawing groups generally enhanced the efficiency of the one-pot sequence. Both chloro substrates 1d and 1e gave consistently higher yields than what has previously been reported.^15,20^ Strongly withdrawing or polar substituents, such as the cyano (1g) and ester (1h) groups, were also well tolerated, indicating that electron-deficient substrates are particularly well suited to the metal-free conditions.

The brominated substrates 1f, 1i, and 1k all underwent smooth annulation, however debrominated products were also observed. For monobrominated substrates 1f and 1i, the desired bromoindoles were the major products, accompanied by only minor amounts of indole. In contrast, dibrominated 1k gave a more complex mixture in which partial debromination was markedly more pronounced. Notably, no debromination was observed for 1l, which gave 6-bromo-5-methoxyindole (3l), a key intermediate in the total synthesis of breitfussin C, G, and H, in good yield.

### Reductive debromination

We found that 1f gave both 5-bromoindole (3f) and indole in 64–68% and 5–7% isolated yields, respectively, while 1i gave a mixture of 6-bromoindole (3i, 53–58%) and indole (5–7%). Further, the dibrominated substrate 1k gave a mixture consisting of 5,6-dibromoindole (3k, 32–34%), 5-bromoindole (10–11%), and 6-bromoindole (5–16%).

To our knowledge, the Leimgruber–Batcho condensation and related protocols have not previously been reported to give reductive debromination, with multiple reported preparations of 6-bromoindole (3i) from substrate 1i.^15,20,25^ We therefore decided to investigate the outcome of each reaction step more closely using GC-MS. Under our conditions, the condensation step furnished the brominated enamine intermediate from 1i and debrominated material in trace (<1%) or non-detectable amounts by GC-MS, indicating that debromination occurs in the reductive annulation step.

**Scheme 3:**
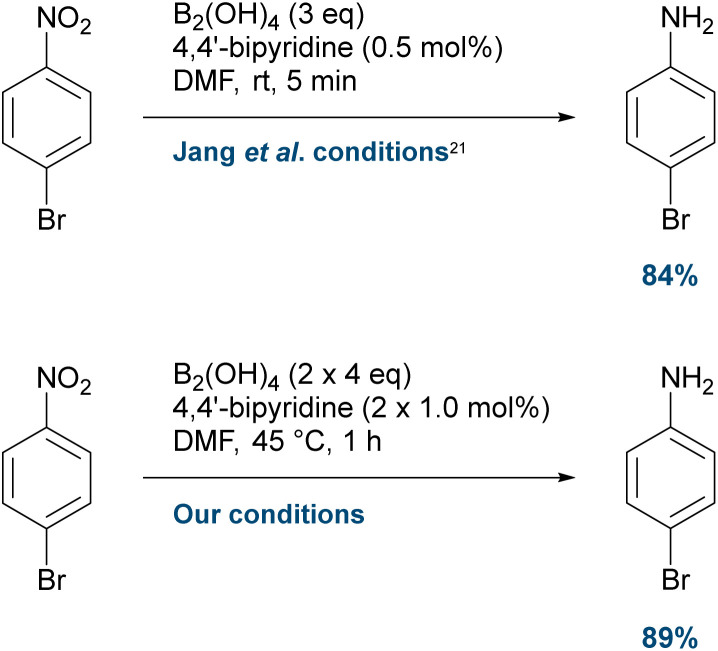
Control reductions of 1-bromo-4-nitrobenzene. No debromination observed.

To ensure that the observed debromination was not an artefact of reagent quality or experimental setup, we first repeated the reduction of 1-bromo-4-nitrobenzene as reported by Jang *et al.* (Scheme 3).^21^ No debromination was detectable by GC-MS, analysis, which is consistent with what was reported,^21^ and 4-bromoaniline was obtained in 84% isolated yield, along with 10% recovered starting material. When 4-bromoaniline was submitted to our refined reduction conditions, complete conversion to the aniline was observed by GC-MS, and the product was isolated in 89% yield. No traces of debromination could be observed by GC-MS analysis.

**Fig. 2:**
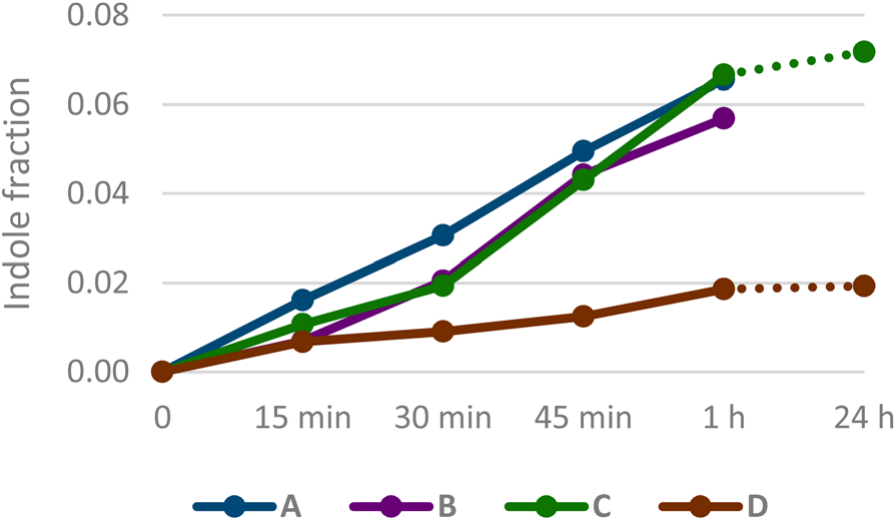
Indole fraction formed during the reduction step under various conditions (A–C) and in a control (D). Quantification was based on GC-MS analysis after each of four sequential additions of reductant. The final data points for experiments (C) and (D) correspond to the analysis of the reaction mixtures after 24 h.

Next, we examined the reductive annulation step more closely by conducting four experiments with GC-MS monitoring. Three experiments employed substrate 1i in a two-step sequence in which the condensation step was kept identical to those presented in Table 2 (4.0 mmol scale), while the conditions of the reduction step were varied. To monitor the onset and progression of the debromination, the reductant and catalyst were added in four portions (4 × 2 equivalents B_2_(OH)_4_, 4 × 0.5 mol% 4,4′-bipyridine), each followed by 15 min of stirring before a sample was taken for GC-MS analysis (Fig. 2). Experiment (A) reproduced the standard reduction conditions used in Table 2, with the only modification being the stepwise addition. Experiment (B) was conducted at room temperature using freshly opened batches of both reductant and catalyst from a different supplier. For experiment (C), the reaction mixture from the condensation step was evaporated to dryness to remove excess reagents, yielding a crimson residue corresponding to the enamine intermediate. This material was redissolved in DMF and subjected to reduction at room-temperature using the same reagent sources as in experiment (B). In experiment (D), commercially sourced 6-bromoindole (3i) was subjected directly to the reduction conditions at room temperature.

Calibration curves were established for indole and 6-bromoindole to enable quantitative GC-MS analysis. The relative response factor (RRF) of indole to 6-bromoindole was determined to be 1.7.

Interestingly, experiment (A–C) gave very similar product distribution (Fig. 2). Debromination was observed after the first aliquot of reductant had been added, and the amount was increased incrementally with each additional aliquot to give around 6–7% of indole compared to 6-bromoindole. Submitting commercially sourced 6-bromoindole to the conditions gave around 2% of indole. Stirring for up to 24 h did not result in any significant change in product distribution.

While Jang *et al.* showed that radical-trapping experiments did not indicate a significant radical contribution to the reduction of nitroarenes under their B_2_(OH)_4_/4,4′-bipyridine conditions,^21^ their study focused on electron-deficient nitroaromatic substrates, whereas the brominated intermediates in our system are electron-rich enamines with markedly different electronic properties. Studies on related diboron-pyridine systems have demonstrated that low-level radical intermediates can arise during diboron activation,^26,27^ and aryl bromides are known to undergo single-electron-driven reductive cleavage even under mild and metal-free conditions.^28^ In the context of this work and our reproducible observation of debromination, a plausible explanation is that a minor, substrate-dependent SET pathway operates alongside the main reduction sequence and selectively affects the C–Br bond. The extent of debromination is modest, and its complete absence for substrate 1l indicates that the electronic environment of the substrate plays a central role in modulating this side reaction.

## Experimental

### General procedure for 2,3-unsubstituted indoles

A dry 100 mL round-bottom flask equipped with a magnetic stir bar was flushed with argon and charged with the appropriate *o*-nitrotoluene 1a–l (4.0 mmol), DMF-DMA (8.0 mmol), pyrrolidine (8.0 mmol), and DMF (5 mL). The flask was fitted with a reflux condenser, and the mixture was heated at 110 °C for 2 h. The resulting crimson-red solution was cooled to 45 °C and diluted with DMF (15 mL). Tetrahydroxydiboron (16 mmol) and 4,4′-bipyridine (1.0 mol%) were then added under ambient atmosphere. After stirring at 45 °C for 30 min, a second portion of tetrahydroxydiboron (16 mmol) and 4,4′-bipyridine (1.0 mol%) was added. Stirring was continued for 30 min, after which TLC analysis indicated complete conversion of the starting material. The reaction mixture was separated between ethyl acetate (100 mL) and water (100 mL), and the aqueous layer was extracted with ethyl acetate (2 × 50 mL). The combined organic layers were dried over Na_2_SO_4_, filtered and concentrated *in vacuo*. The crude product was purified by flash column chromatography on silica gel using a mixture of ethyl acetate and petroleum ether as the eluent, affording the desired indole products 3a–l.

## Conclusions

In summary, we have developed a concise, metal-free, one-pot protocol for the synthesis of 2,3-unsubstituted indoles as an alternative to the classical Leimgruber–Batcho protocol. By incorporating a mild, bipyridine-catalysed reduction with tetrahydroxydiboron, the method avoids the use of transition metals and hazardous reductants. The protocol exhibits broad substrate scope and produces indoles from electronically and sterically demanding substrates in moderate to good yields. Its utility was further illustrated by the direct synthesis of 6-bromo-5-methoxyindole, a key intermediate in the total syntheses of breitfussin C, G, and H. Low-level substrate-dependent debromination was observed for certain brominated derivatives, although this effect was absent for electronically stabilised systems. Given its efficiency, simplicity, and scalability, this protocol should be well-suited for the preparation of 2,3-unsubstituted indoles.

## Author contributions

Conceptualisation: BEH; investigation and methodology: BH; supervision: BEH; writing – original draft: BH; writing – review and editing: BH, BEH.

## Conflicts of interest

The authors declare no conflict of interest.

## Supplementary Material

RA-016-D6RA02702D-s001

## Data Availability

The data supporting this article are included in the supplementary information (SI). Supplementary information is available. See DOI: https://doi.org/10.1039/d6ra02702d.
