# Supplementary material for: Application of metal-free conditions to a one-pot Leimgruber–Batcho indole synthesis
Source: RSC Adv. 2026 May 19;16(29):26858–63. doi: 10.1039/d6ra02702d (PMC13187892; doi:10.1039/d6ra02702d)
Supplement: RA-016-D6RA02702D-s001 [file RA-016-D6RA02702D-s001.pdf]

Supplementary Information (SI) for RSC Advances.
This journal is © The Royal Society of Chemistry 2026

Supplementary Information

Application of metal-free conditions to a one-pot Leimgruber–Batcho indole synthesis

Beate Halsvik and Bengt Erik Haug*

University of Bergen, Department of Chemistry and Centre for Pharmacy, Allégaten 41, 5020 Bergen, Norway

*To whom correspondence should be addressed.

Tel: +47 55583468. E-mail: bengt-erik.haug@uib.no

Table of Contents

Experimental 2

General 2

Procedures 3

NMR spectra 9

References 37

Experimental

General

Chemicals

All chemicals were purchased from Sigma-Aldrich and used without any purification unless otherwise stated. All anhydrous solvents were used as delivered. Tetrahydroxydiboron was sourced from multiple suppliers, depending on availability at the time of use, specifically from TCI Europe, BLD Pharm and Fluorochem.

The following starting materials were obtained from Aaron Chemicals: 4-chloro-2-methyl-1-nitrobenzene, 4-bromo-2-methyl-1-nitrobenzene, 3-methyl-4-nitrobenzonitrile, methyl 3-methyl-4-nitrobenzoate, 4-bromo-1-methyl-2-nitrobenzene and 1,2-dibromo-4-methyl-5-nitrobenzene.

Experimental descriptions

All reactions performed under dry conditions were carried out in oven-dried glassware (130 °C) equipped with a septum under an argon atmosphere, using dry solvents (see details above).

Chromatography

Thin-layer chromatography (TLC) analyses were performed on aluminium-backed silica gel plates (60 F_{254}). Eluent systems consisted of mixtures of petroleum ether and ethyl acetate, selected based on compound polarity. TLC spots were visualised using UV light at 254 nm.

Flash column chromatography was conducted using an Interchim puriFlash® XS520Plus system equipped with a multi-wavelength UV-DAD detector and a quaternary gradient pump.

NMR Spectroscopy

NMR spectroscopic analyses were performed on a Bruker BioSpin AVANCE NEO 600 spectrometer with the field strength of 600 MHz for ^{1}H nuclei and 151 MHz for ^{13}C nuclei. Deuterated chloroform ($CDCl_3$) was used as the solvent. Chemical shifts (δ) are reported in parts per million (ppm) relative to the residual solvent peak (δ_{1H} = 7.26 ppm, δ_{13C} = 77.2 ppm). Coupling constants (*J*) are given in Hertz (Hz), and signal multiplicities are designated as follows: (s) singlet, (d) doublet, (t) triplet, (m) multiplet, and (dd) doublet of doublets.

Procedures

General procedure for 2,3-unsubstituted indoles.

A dry 100 mL round-bottom flask equipped with a magnetic stir bar was flushed with argon and charged with the appropriate *o*-nitrotoluene **1a-l** (4.0 mmol), DMF-DMA (8.0 mmol), pyrrolidine (8.0 mmol), and DMF (5 mL). The flask was fitted with a reflux condenser, and the mixture was heated at 110 °C for 2 h. The resulting crimson-red solution was cooled to 45 °C and diluted with DMF (15 mL). Tetrahydroxydiboron (16 mmol) and 4,4′-bipyridine (1.0 mol%) were then added under ambient atmosphere. After stirring at 45 °C for 30 min, a second portion of tetrahydroxydiboron (16 mmol) and 4,4′-bipyridine (1.0 mol%) was added. Stirring was continued for 30 min, after which TLC analysis indicated complete conversion of the starting material. The reaction mixture was separated between ethyl acetate (100 mL) and water (100 mL), and the aqueous layer was extracted with ethyl acetate (2 × 50 mL). The combined organic layers were dried over Na_2SO_4, filtered and concentrated *in vacuo*. The crude product was purified by flash column chromatography on silica gel using a mixture of ethyl acetate and petroleum ether as the eluent, affording the desired indole products **3a-l**.

1-Bromo-2-methoxy-4-methyl-5-nitrobenzene (1l):

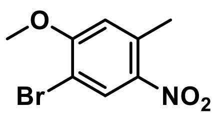

To a solution of 1-bromo-2-fluoro-4-methyl-5-nitrobenzene (2.26 g, 9.7 mmol) in dry methanol (40 mL) was added sodium methoxide (3.26 g, 60 mmol), and the resulting mixture was stirred at 30 °C for 48 h, upon which TLC analysis showed full conversion of the starting material. The reaction was quenched by adding 10% Na_2CO_3 (15 mL). The resulting thick suspension was diluted with cold water (20 mL) and filtered using a Büchner funnel. The solid residue was washed with cold ethyl acetate (5 mL). The solid residue was suspended in ethyl acetate (100 mL), washed with water (100 mL), dried over Na_2SO_4, filtered, and concentrated *in vacuo* to give a light-yellow solid (1.86 g). An additional portion of the product (440 mg) was obtained by diluting the initial filtrate with saturated NaCl (30 mL), extracting with ethyl acetate (3 × 25 mL), washing the combined organic layers with water (30 mL) and saturated NaCl (30 mL), drying over Na_2SO_4, filtering, and concentrating *in vacuo*. Combining the two batches gave a light-yellow solid (2.30 g, 97%), which was used without further purification. R_F = 0.77 (EtOAc/pet. ether, 1:1 v/v); ^{1}H NMR (600 MHz, $CDCl_3$): δ = 8.34 (s, 1H), 6.76 (s, 1H), 3.98 (s, 3H), 2.64 (s, 3H); ^{13}C NMR (151 MHz, $CDCl_3$): δ = 159.5, 142.2, 136.4, 130.6, 114.6, 109.1, 56.9, 21.8;

HRMS (EI-TOF+): *m/z* $[M+H]^{+\bullet}$ calcd for $C_8H_9BrNO_3^{+\bullet}$ 244.9683/246.9662; found 244.9688/246.9667. The analytical data were consistent with previously reported values.[1]

Indole (3a):

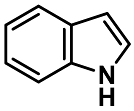

The title compound was prepared from 1-methyl-2-nitrobenzene (549 mg, 4.0 mmol) following the general procedure to give a light-yellow solid (359 mg, 77%); R_F = 0.82 (EtOAc/pet. ether, 1:1 v/v); ^{1}H NMR (600 MHz, $CDCl_3$): δ = 8.07 (s, 1H), 7.70 (d, *J* = 7.9, 1H), 7.41 (d, *J* = 8.2, 1H), 7.27 – 7.21 (m, 1H), 7.20 (t, *J* = 2.8, 1H), 7.20 – 7.15 (m, 1H), 6.60 (t, *J* = 2.7, 1H); ^{13}C NMR (151 MHz, $CDCl_3$): δ = 135.9, 128.0, 124.3, 122.1, 120.8, 119.9, 111.1, 102.7; HRMS (EI-TOF+): *m/z* $[M]^{+\bullet}$ calcd for $C_8H_7N^{+\bullet}$ 117.0573; found 117.0598. The analytical data were consistent with previously reported values.[2]

5-Methylindole (3b):

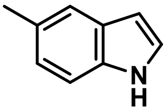

The title compound was prepared from 2,4-dimethyl-1-nitrobenzene (605 mg, 4.0 mmol) following the general procedure to give a colourless solid (234 mg, 44%); R_F = 0.74 (EtOAc/pet. ether, 1:1 v/v); ^{1}H NMR (600 MHz, $CDCl_3$): δ = 7.93 (s, 1H), 7.52 (s, 1H), 7.30 (d, *J* = 8.3, 1H), 7.15 (t, *J* = 2.8, 1H), 7.10 (d, *J* = 8.3, 1H), 6.54 (s, 1H), 2.53 (s, 3H); ^{13}C NMR (151 MHz, $CDCl_3$): δ = 134.2, 129.1, 128.2, 124.4, 123.7, 120.4, 110.8, 102.1, 21.5; HRMS (EI-TOF+): *m/z* $[M]^{+\bullet}$ calcd for $C_9H_9N^{+\bullet}$ 131.0730; found 131.0735. The analytical data were consistent with previously reported values.[2]

4-Aminoindole (3c):

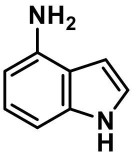

The title compound was prepared from 2-methyl-3-nitroaniline (609 mg, 4.0 mmol) following the general procedure to give a dark grey solid (239 mg, 45%); R_F = 0.53 (EtOAc/pet. ether, 1:1 v/v); ^{1}H NMR (600 MHz, $CDCl_3$): δ = 8.11 (s, 1H), 7.11 (t, *J* = 2.9, 1H), 7.03 (t, *J* = 7.8, 1H), 6.88 (dd, *J* = 8.1, 1.0, 1H), 6.50 – 6.46 (m, 1H), 6.42 (d, *J* = 7.4, 1H), 3.93 (s, 2H); ^{13}C NMR (151 MHz, $CDCl_3$): δ = 139.5, 137.0, 123.3, 122.5, 117.5, 104.3, 102.3, 99.0; HRMS (EI-TOF+): *m/z* $[M]^{+\bullet}$ calcd for $C_8H_8N_2^{+\bullet}$ 132.0682; found 132.0687. The analytical data were consistent with previously reported values.[2]

4-Chloroindole (3d):

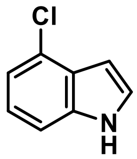

The title compound was prepared from 1-chloro-2-methyl-3-nitrobenzene (686 mg, 4.0 mmol) following the general procedure to give a yellow oil (402 mg, 67%); R_F = 0.70 (EtOAc/pet. ether, 1:1 v/v); ^{1}H NMR (600 MHz, $CDCl_3$): δ = 8.25 (s, 1H), 7.30 (d, *J* = 7.5, 1H), 7.25 (t, *J* = 2.8, 1H), 7.16 – 7.09 (m, 2H), 6.68 (t, *J* = 2.7, 1H); ^{13}C NMR (151 MHz, $CDCl_3$): δ = 136.6, 126.9, 126.3, 124.8, 122.7, 119.7, 109.8, 101.5; HRMS (EI-TOF+): *m/z* $[M]^{+\bullet}$ calcd for $C_8H_6ClN^{+\bullet}$ 151.0183; found 151.0186. The analytical data were consistent with previously reported values.[2]

5-Chloroindole (3e):

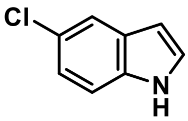

The title compound was prepared from 4-chloro-2-methyl-1-nitrobenzene (690 mg, 4.0 mmol) following the general procedure to give a beige solid (499 mg, 82%); R_F = 0.76 (EtOAc/pet. ether, 1:1 v/v); ^{1}H NMR (600 MHz, $CDCl_3$): δ = 8.18 (s, 1H), 7.62 (d, *J* = 2.1, 1H), 7.30 (d, *J* = 8.6, 1H), 7.23 (t, *J* = 2.9, 1H), 7.16 (dd, *J* = 8.7, 1.5, 1H), 6.51 (m, 1H); ^{13}C NMR (151 MHz, $CDCl_3$): δ = 134.3, 129.1, 125.7, 125.6, 122.4, 120.2, 112.1, 102.5; HRMS (EI-TOF+): *m/z* $[M]^{+\bullet}$ calcd for $C_8H_6ClN^{+\bullet}$ 151.0189; found 151.0187. The analytical data were consistent with previously reported values.[2]

5-Bromoindole (3f):

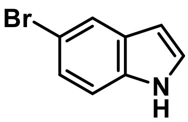

The title compound was prepared from 4-bromo-2-methyl-1-nitrobenzene (863 mg, 4.0 mmol) following the general procedure. Purification by column chromatography gave the title compound (353 mg, 45%) in addition to a mixture of the title compound and indole (24 mg (5%) indole and 180 mg (23%) 5-bromoindole by ^{1}H NMR analysis); R_F = 0.74 (EtOAc/pet. ether, 1:1 v/v); ^{1}H NMR (600 MHz, $CDCl_3$): δ = 8.18 (s, 1H), 7.78 (d, *J* = 1.8, 1H), 7.30 – 7.26 (m, 2H), 7.21 (t, *J* = 2.7, 1H), 6.50 (m, 1H); ^{13}C NMR (151 MHz, $CDCl_3$): δ = 134.5, 129.8, 125.5, 125.0, 123.4, 113.2, 112.6, 102.4; HRMS (EI-TOF+): *m/z* $[M]^{+\bullet}$ calcd for $C_8H_6BrN^{+\bullet}$ 194.9684/196.9663; found 194.9679/196.9656. The analytical data were consistent with previously reported values.[3]

5-Cyanoindole (3g):

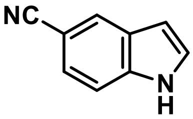

The title compound was prepared from 3-methyl-4-nitrobenzonitrile (652 mg, 4.0 mmol) following the general procedure to give a beige solid (431 mg, 76%); R_F = 0.57 (EtOAc/pet. ether, 1:1 v/v); ^{1}H NMR (600 MHz, CDCl3); δ = 8.53 (s, 1H), 8.00 (s, 1H), 7.48 – 7.41 (m, 2H), 7.36 – 7.32 (m, 1H), 6.66 – 6.62 (m, 1H); ^{13}C NMR (151 MHz, $CDCl_3$): δ = 137.6, 127.8, 126.6, 126.5, 125.1, 120.9, 112.1, 103.7, 103.2; HRMS (EI-TOF+): *m/z* $[M]^{+\bullet}$ calcd for $C_9H_6N_2^{+\bullet}$ 142.0531; found 142.0511. The analytical data were consistent with previously reported values.[2]

Methyl indole-5-carboxylate (3h)

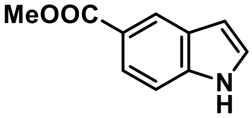

The title compound was prepared from methyl 3-methyl-4-nitrobenzoate (788 mg, 4.0 mmol) following the general procedure to give a beige solid (518 mg, 74%); R_F = 0.61 (EtOAc/pet. ether, 1:1 v/v); ^{1}H NMR (600 MHz, CDCl3); δ = 8.44 – 8.40 (m, 2H), 7.91 (dd, *J* = 8.6, 1.6, 1H), 7.41 (d, *J* = 8.6, 1H), 7.27 (t, *J* = 3.3, 1H), 6.67 – 6.63 (m, 1H), 3.94 (s, 3H); ^{13}C NMR (151 MHz, $CDCl_3$): δ = 168.4, 138.5, 127.6, 125.6, 123.9, 123.5, 122.1, 110.9, 104.2, 52.0; HRMS (EI-TOF+): *m/z* $[M]^{+\bullet}$ calcd for $C_{10}H_9NO_2^{+\bullet}$ 175.0633; found 175.0630. The analytical data were consistent with previously reported values.[4]

6-Bromoindole (3i):

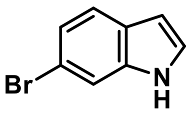

The title compound was prepared from 4-bromo-1-methyl-2-nitrobenzene (859 mg, 4.0 mmol) using the general procedure, yielding a brown solid containing a mixture of indole (32 mg, 7%) and 6-bromoindole (449 mg, 58%). Yields were determined by ^{1}H NMR analysis. A portion of the material was further purified, allowing isolation of 6-bromoindole in analytically pure form (21 mg) for full characterisation; R_F = 0.80 (EtOAc/pet. ether, 1:1 v/v); ^{1}H NMR (600 MHz, CDCl3): δ = 7.98 (s, 1H), 7.50 – 7.44 (m, 2H), 7.21 (dd, *J* = 8.4, 1.8, 1H), 7.10 (t, *J* = 3.3, 1H), 6.52 – 6.48 (m, 1H); ^{13}C NMR (151 MHz, $CDCl_3$): δ = 136.6, 126.8, 124.9, 123.2, 122.0, 115.5, 114.1, 102.9; HRMS (EI-TOF+): *m/z* $[M]^{+\bullet}$ calcd for $C_8H_6BrN^{+\bullet}$ 194.9684/196.9663; found 194.9680/196.9658. The analytical data were consistent with previously reported values.[2]

7-Methylindole (3j):

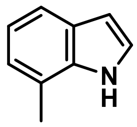

The title compound was prepared from 1,3-dimethyl-2-nitrobenzene (605 mg, 4.0 mmol) following the general procedure to give a colourless solid (221 mg, 42%); R_F = 0.75 (EtOAc/pet. ether, 1:1 v/v); ^{1}H NMR (600 MHz, $CDCl_3$): δ = 8.05 (s, 1H), 7.54 (d, *J* = 7.8, 1H), 7.21 (t, *J* = 2.8, 1H), 7.08 (t, *J* = 7.5, 1H), 7.03 (d, *J* = 7.6, 1H), 6.60 (dd, *J* = 3.2, 2.0, 1H), 2.52 (s, 3H); ^{13}C NMR (151 MHz, $CDCl_3$): δ = 135.5, 127.5, 123.9, 122.6, 120.3, 120.1, 118.6, 103.2, 16.8; HRMS (EI-TOF+): *m/z* $[M]^{+\bullet}$ calcd for $C_9H_9N^{+\bullet}$ 131.0730; found 131.0721. The analytical data were consistent with previously reported values.[2]

5,6-Dibromoindole (3k):

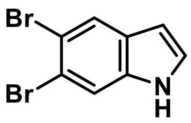

The title compound was prepared from 1,2-dibromo-4-methyl-5-nitrobenzene (1179 mg, 4.0 mmol) following the general procedure, yielding a dark grey solid containing a mixture of 5-bromoindole (84 mg, 11%), 6-bromoindole (42 mg, 5%) and the desired 5,6-dibromoindole (348 mg, 32%). Yields were determined by ^{1}H NMR analysis. A portion of the material was further purified, allowing isolation of 5,6-dibromoindole in analytically pure form (14 mg) for full characterisation; R_F = 0.66 (EtOAc/pet. ether, 1:1 v/v); ^{1}H NMR (600 MHz, $CDCl_3$): δ = 7.98 (s, 1H), 7.50 – 7.44 (m, 2H), 7.21 (dd, *J* = 8.4, 1.8, 1H), 7.10 (t, *J* = 3.3, 1H), 6.52 – 6.48 (m, 1H); ^{13}C NMR (151 MHz, $CDCl_3$): δ = 135.6, 128.9, 126.2, 125.1, 117.2, 115.8, 115.2, 102.5; HRMS (EI-TOF+): *m/z* $[M]^{+\bullet}$ calcd for $C_8H_5Br_2N^{+\bullet}$ 272.8789/276.8748; found 272.8785/276.8740. The analytical data were consistent with previously reported values.[5]

6-Bromo-5-methoxyindole (3l):

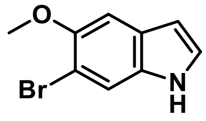

The title compound was prepared from 1-bromo-2-methoxy-4-methyl-5-nitrobenzene (**1l**, 984 mg, 4.0 mmol) following the general procedure to give a light-yellow solid (562 mg, 62%); R_F = 0.75 (EtOAc/pet. ether, 1:1 v/v); ^{1}H NMR (600 MHz, $CDCl_3$): δ = 8.06 (s, 1H), 7.59 (d, *J* = 1.0, 1H), 7.18 (t, *J* = 2.8, 1H), 7.14 (s, 1H), 6.50 – 6.46 (m, 1H), 3.92 (s, 3H); ^{13}C NMR (151 MHz, $CDCl_3$): δ = 150.4, 131.3, 127.8, 125.4, 115.6, 107.5, 102.8, 102.7, 56.9; HRMS (EI-TOF+): *m/z* $[M+H]^{+\bullet}$ calcd for $C_9H_9BrNO^{+\bullet}$ 224.9784/226.9764; found 224.9790/226.9768. The analytical data were consistent with previously reported values.[6]

Control experiment using Jang *et al.*'s reduction conditions.

A dry 10 mL round-bottom flask equipped with a magnetic stir bar was charged with 1-bromo-4-nitrobenzene (303 mg, 1.5 mmol) and DMF (2 mL). Tetrahydroxydiboron (403 mg, 4.5 mmol) and 4,4′-bipyridine (1.3 mg, 0.5 mol%) dissolved in DMF (2 mL) were added under ambient atmosphere. The reaction mixture was stirred at room temperature for 5 min, after which TLC and GC–MS analysis indicated partial conversion with residual starting material present. The reaction mixture was partitioned between ethyl acetate (10 mL) and water (10 mL), and the aqueous layer was extracted with ethyl acetate (2 × 5 mL). The combined organic layers were dried over Na_2SO_4, filtered and concentrated *in vacuo*. The crude product was purified by flash column chromatography on silica gel using a mixture of ethyl acetate and petroleum ether as the eluent, affording 4-bromoaniline as a colourless solid (217 mg, 84%) and recovered 1-bromo-4-nitrobenzene (29 mg, 10%). No debromination products could be detected by GC–MS or NMR analysis.

Control experiment using the refined reduction conditions from the one-pot protocol.

A dry 100 mL round-bottom flask equipped with a magnetic stir bar was charged with 1-bromo-4-nitrobenzene (808 mg, 4.0 mmol) and DMF (20 mL). The mixture was heated to 45 °C, after which tetrahydroxydiboron (1.43 g, 16 mmol) and 4,4′-bipyridine (6 mg, 1.0 mol%) were added under ambient atmosphere. After stirring at 45 °C for 30 min, a second portion of tetrahydroxydiboron (1.44 g, 16 mmol) and 4,4′-bipyridine (6 mg, 1.0 mol%) was added. The reaction was stirred for a further 30 min, at which point TLC and GC–MS analysis indicated complete consumption of the starting material. The reaction mixture was partitioned between ethyl acetate (100 mL) and water (100 mL), and the aqueous layer was extracted with ethyl acetate (2 × 50 mL). The combined organic layers were dried over Na_2SO_4, filtered and concentrated *in vacuo*. The crude product was purified by flash column chromatography on silica gel using a mixture of ethyl acetate and petroleum ether as the eluent, affording 4-bromoaniline as a colourless solid (611 mg, 89%). No debromination products could be detected by GC–MS or NMR analysis.

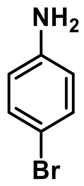

4-bromoaniline: Colourless solid; R_F = 0.54 (EtOAc/pet. ether, 1:1 v/v); ^{1}H NMR (600 MHz, CDCl3): δ = 7.26 – 7.21 (m, 2H), 6.58 – 6.53 (m, 2H), 3.66 (s, 2H); ^{13}C NMR (151 MHz, $CDCl_3$): δ = 145.5, 132.1, 116.8, 110.2. The analytical data were consistent with previously reported values.[7]

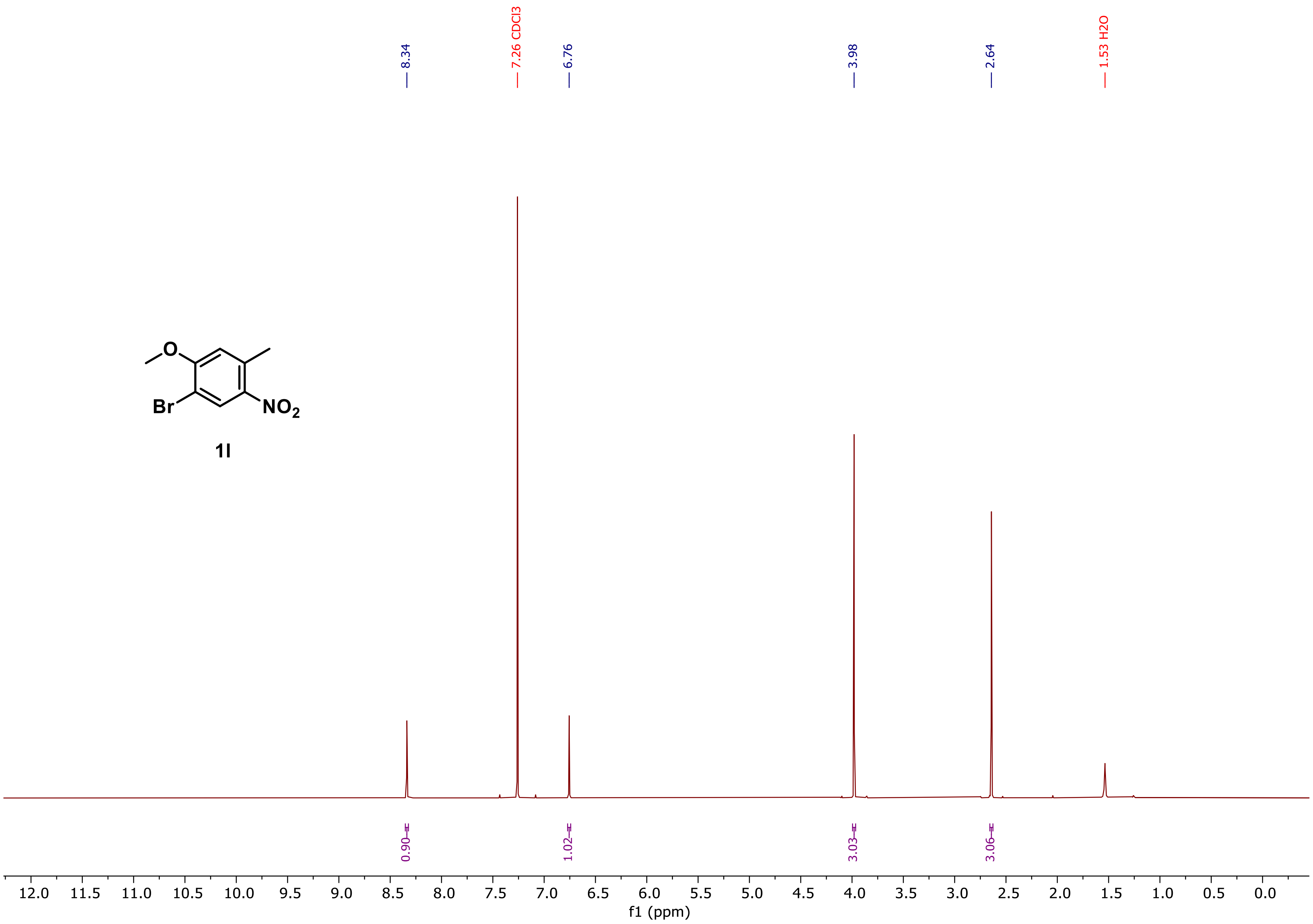

Figure S1: ^{1}H NMR spectrum of **1l**.

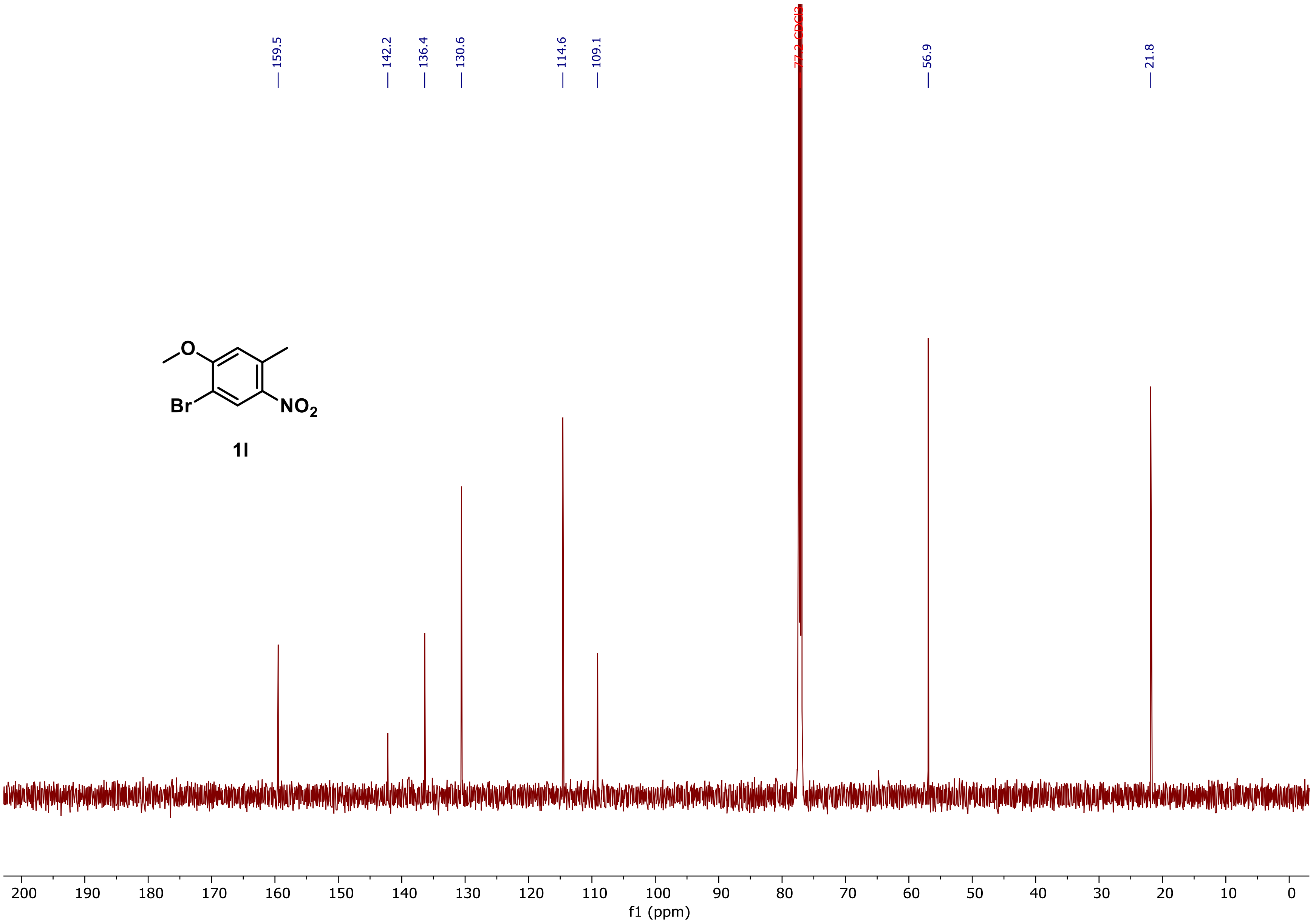

Figure S2: ^{13}C NMR spectrum of **1l**.

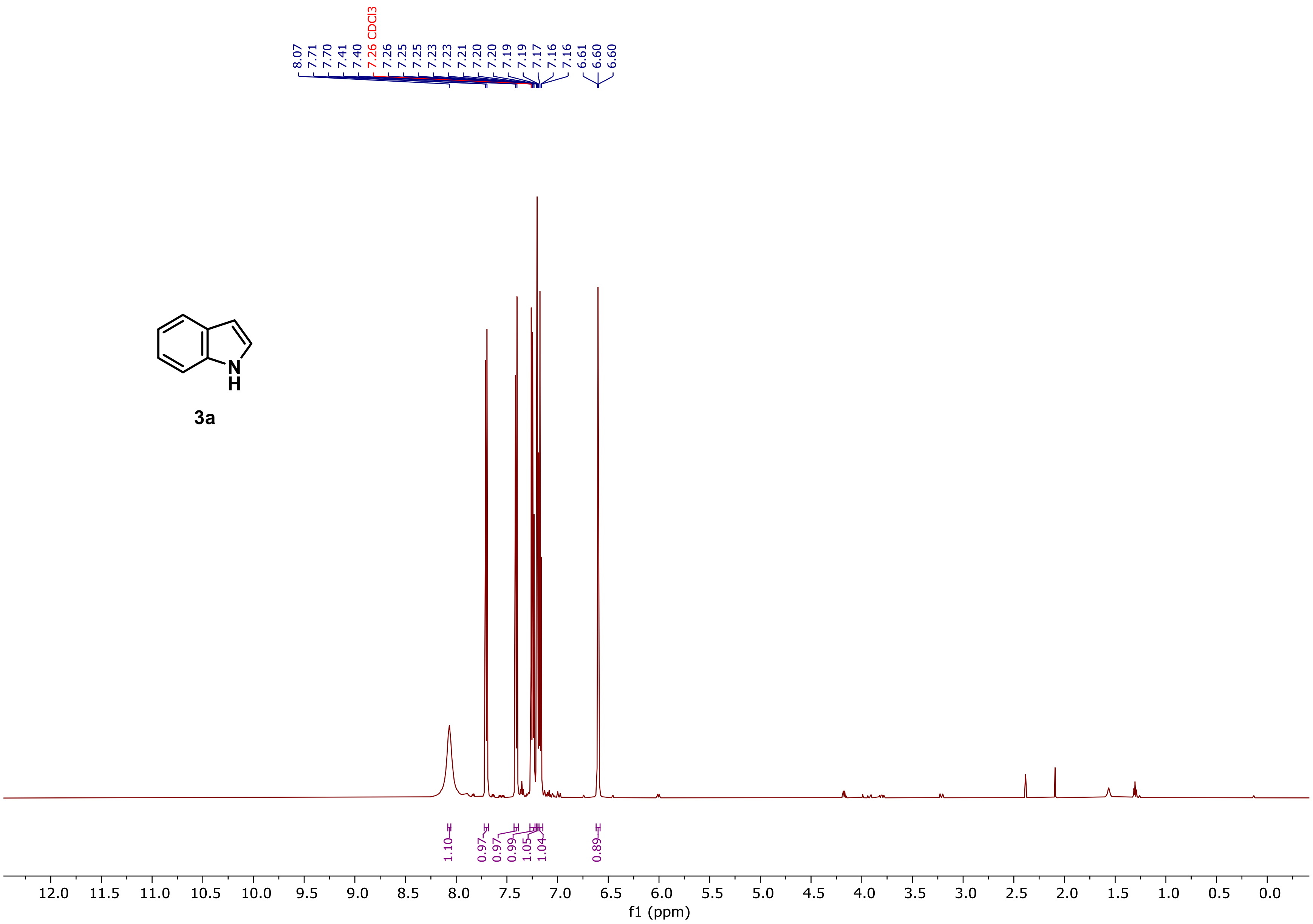

Figure S3: ^{1}H NMR spectrum of **3a**.

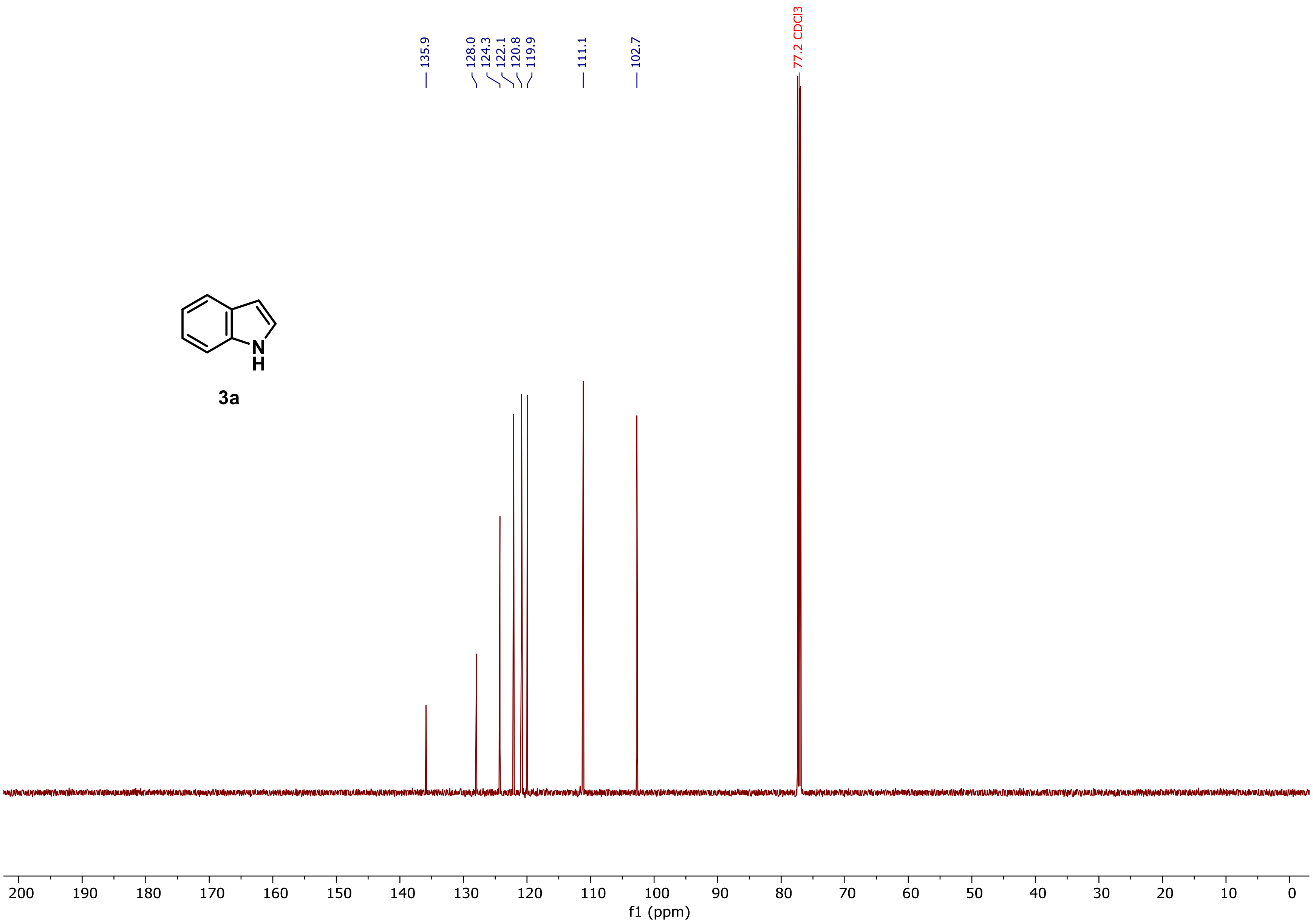

Figure S4: ^{13}C NMR spectrum of **3a**.

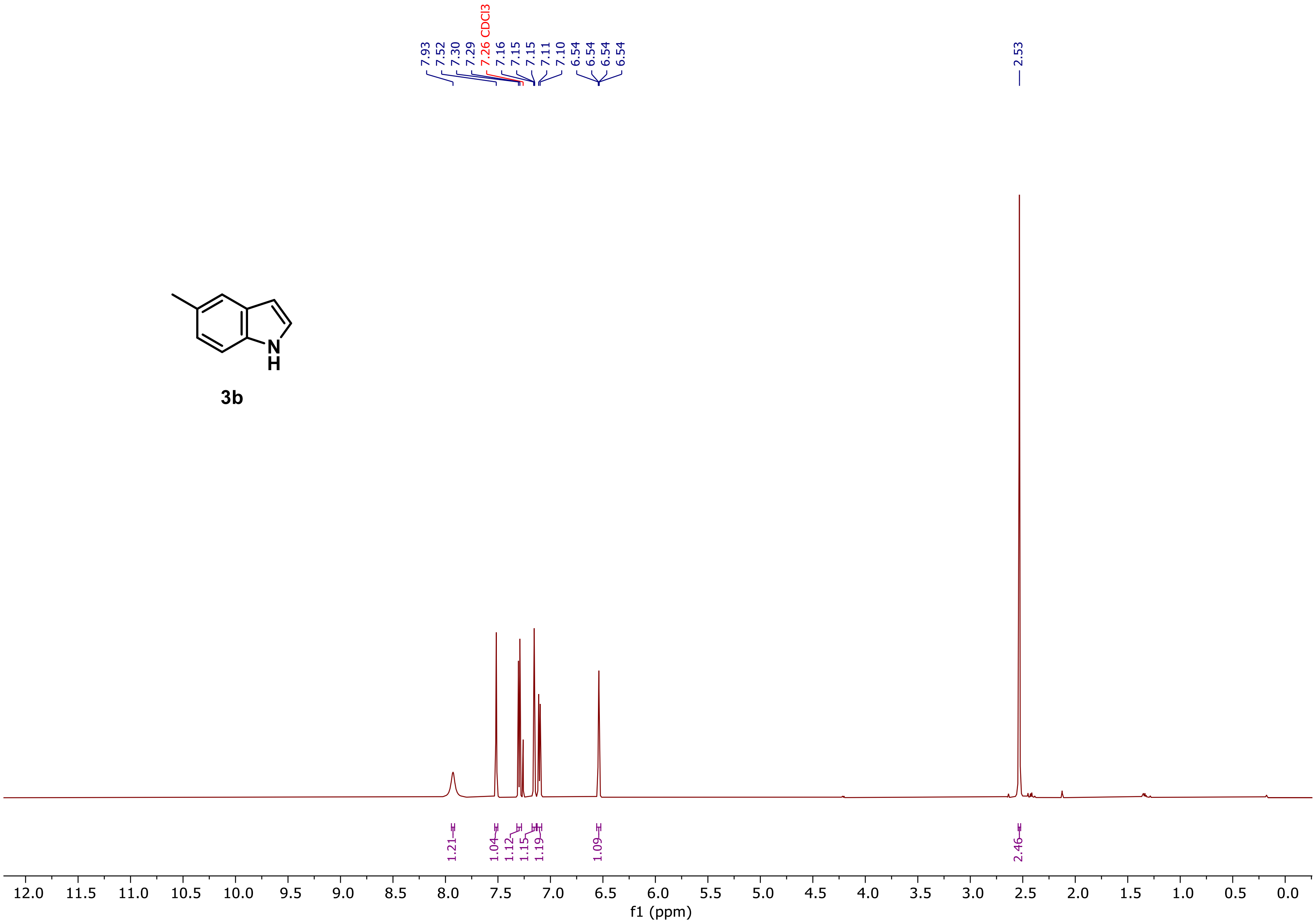

Figure S5: ^{1}H NMR spectrum of **3b**.

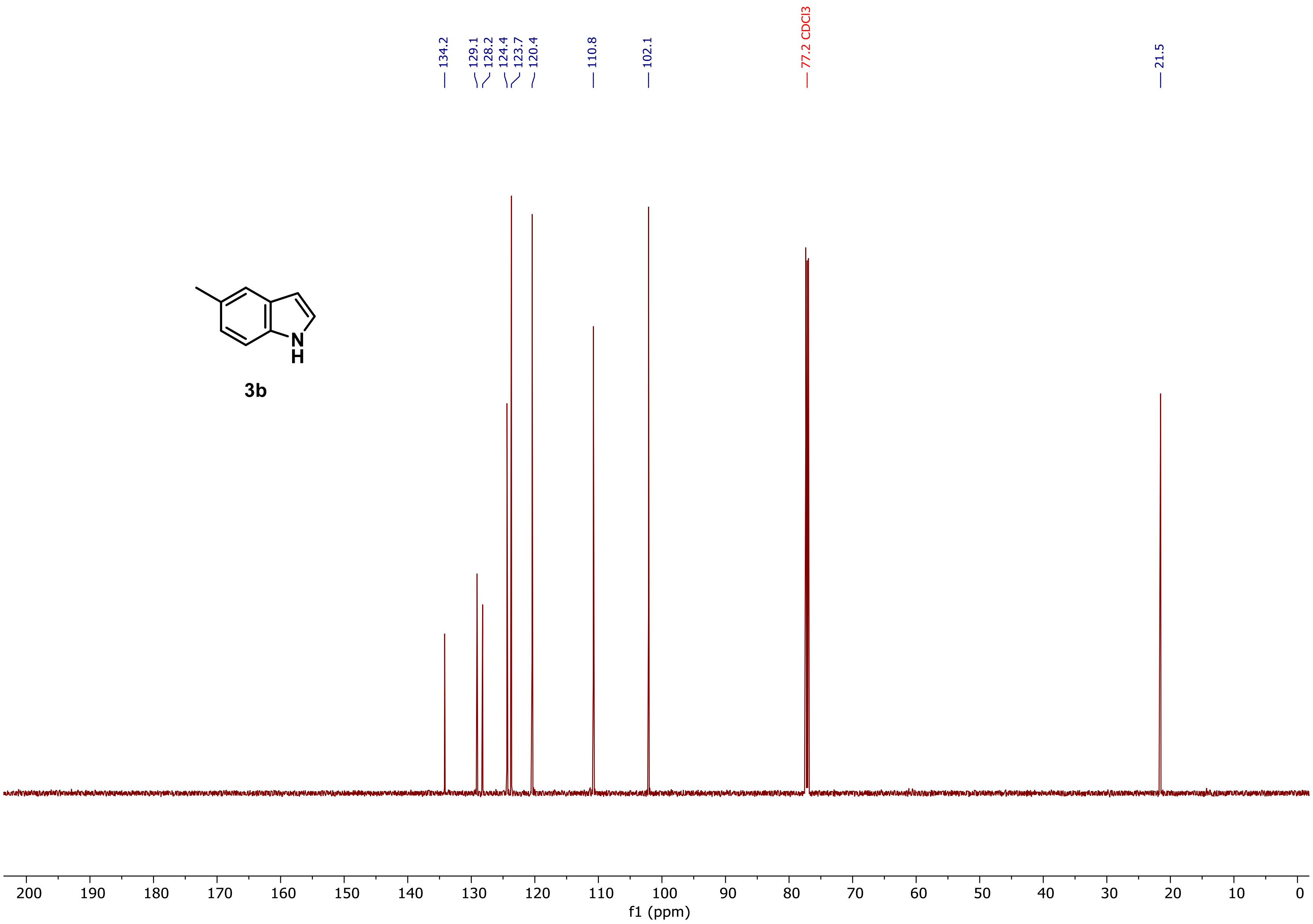

3b

Figure S6: ^{13}C NMR spectrum of **3b**.

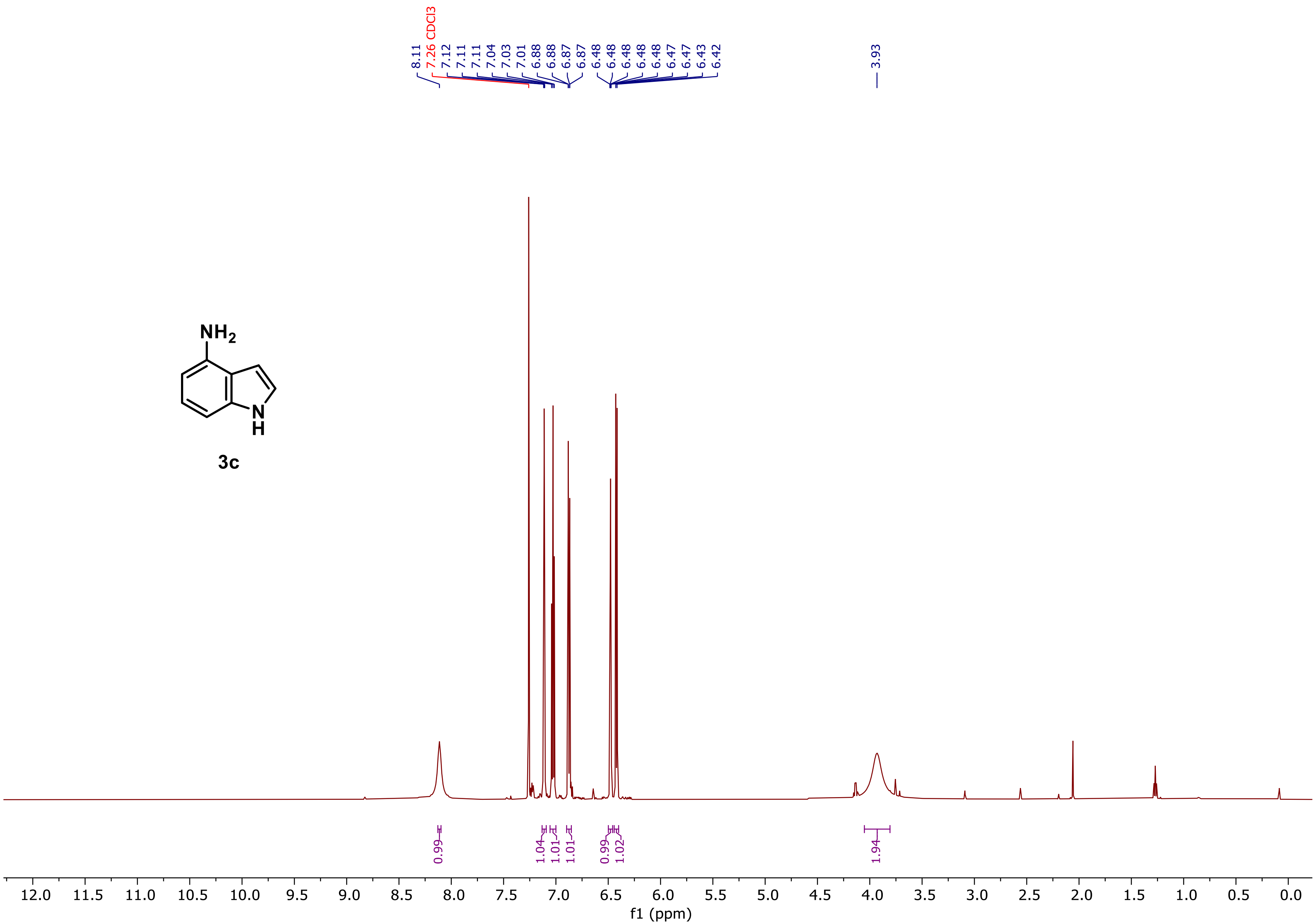

Figure S7: ^{1}H NMR spectrum of **3c**.

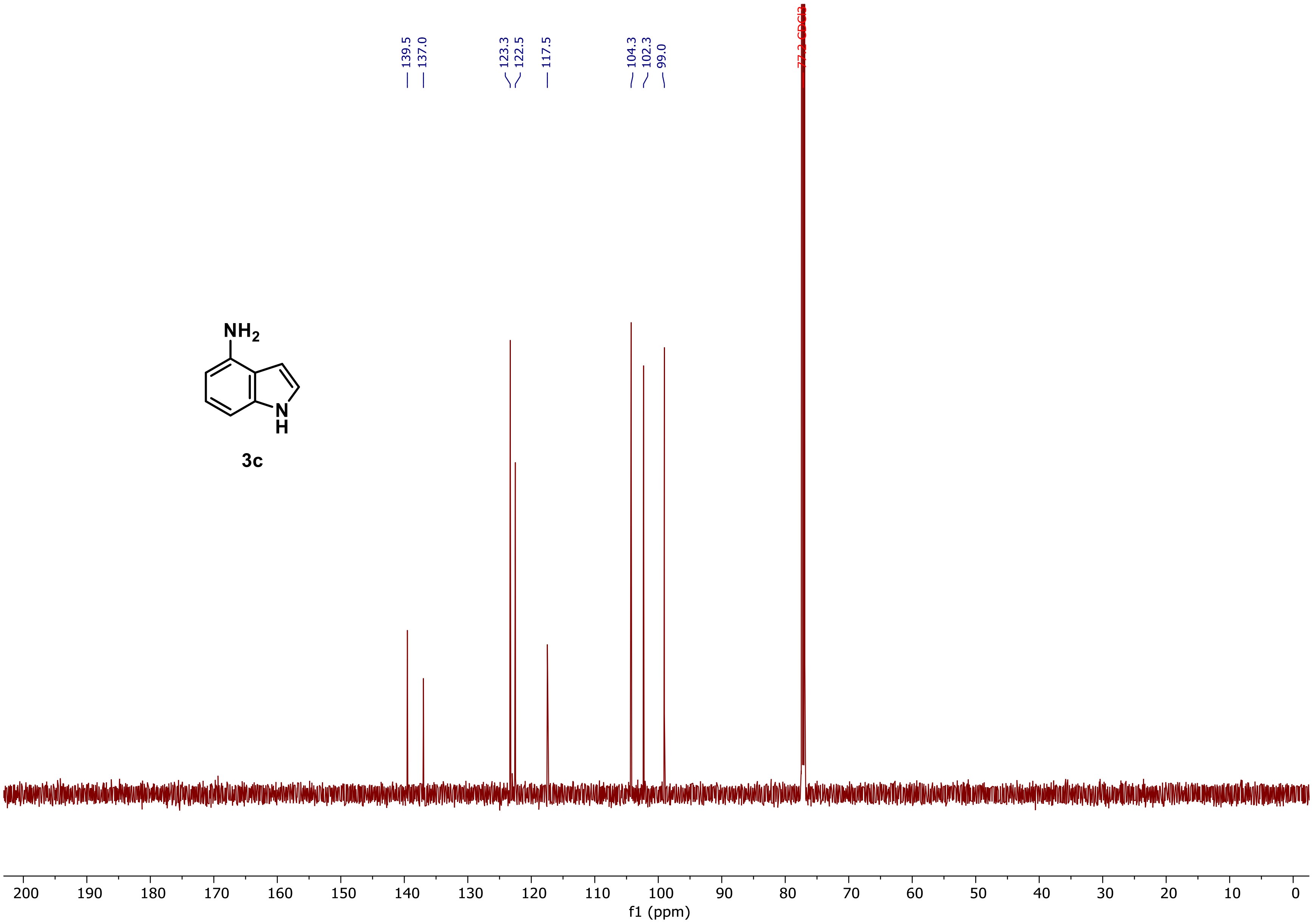

Figure S8: ^{13}C NMR spectrum of **3c**.

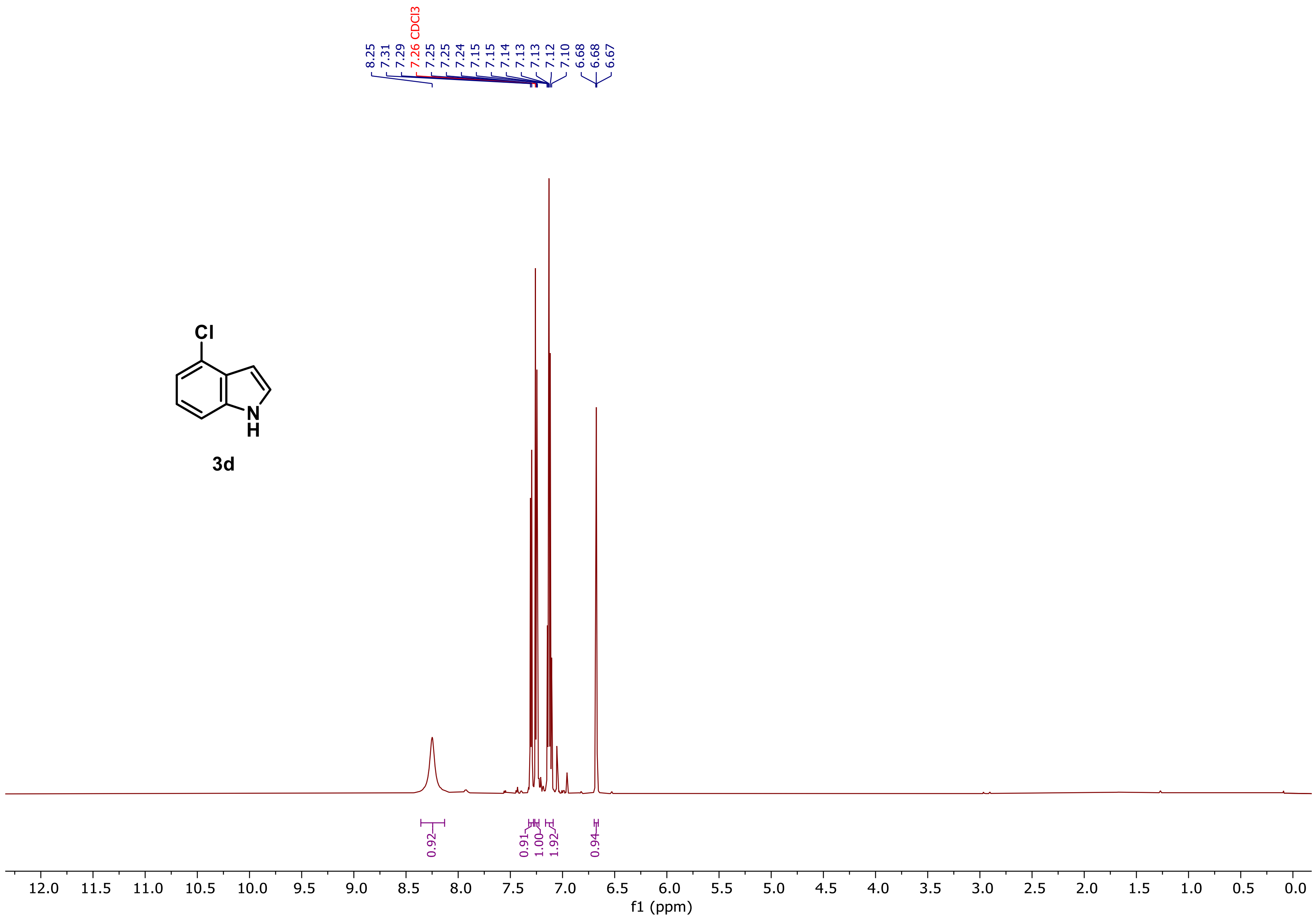

Figure S9: ^{1}H NMR spectrum of **3d**.

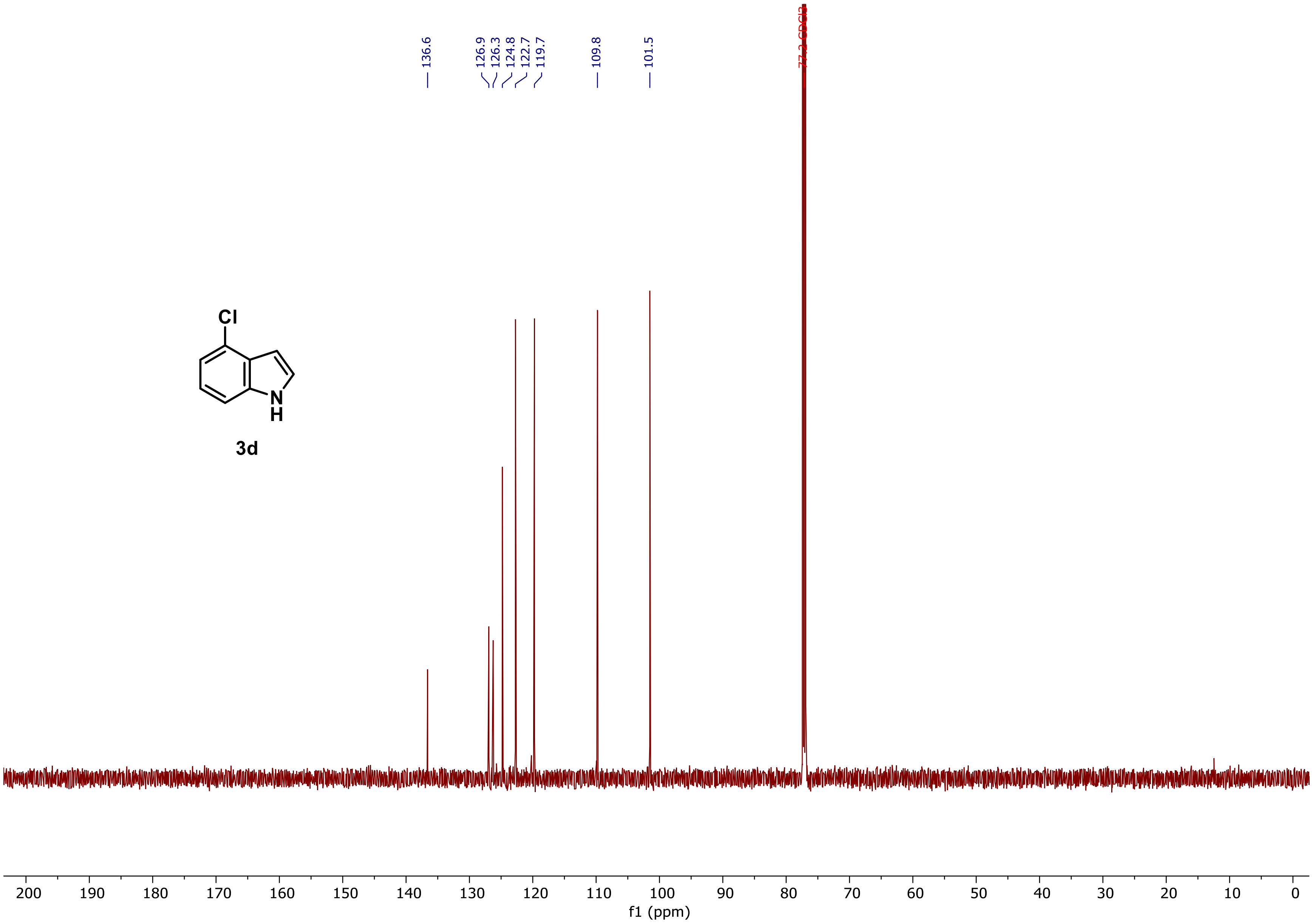

Figure S10: ^{13}C NMR spectrum of **3d**.

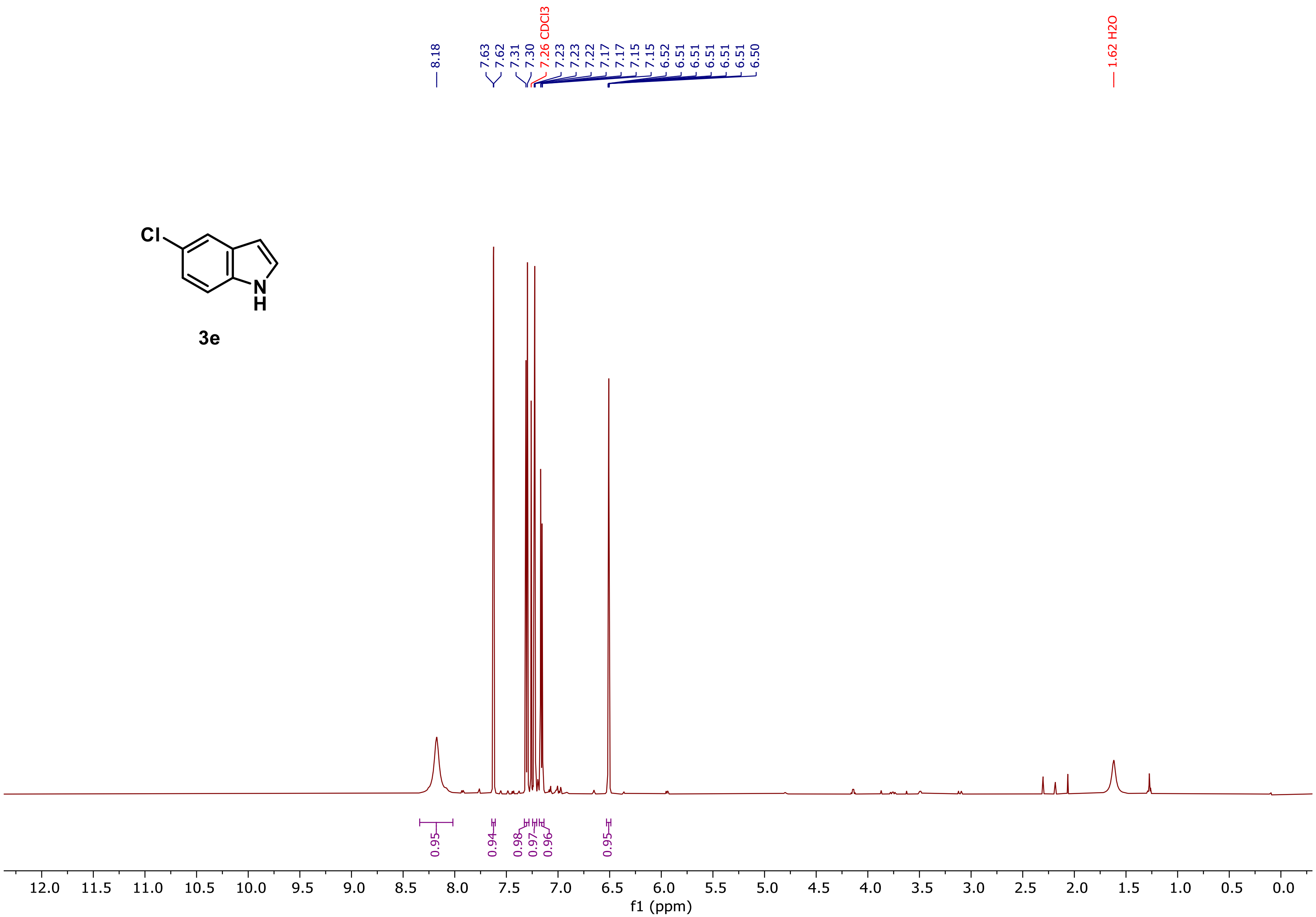

Figure S11: ^{1}H NMR spectrum of **3e**.

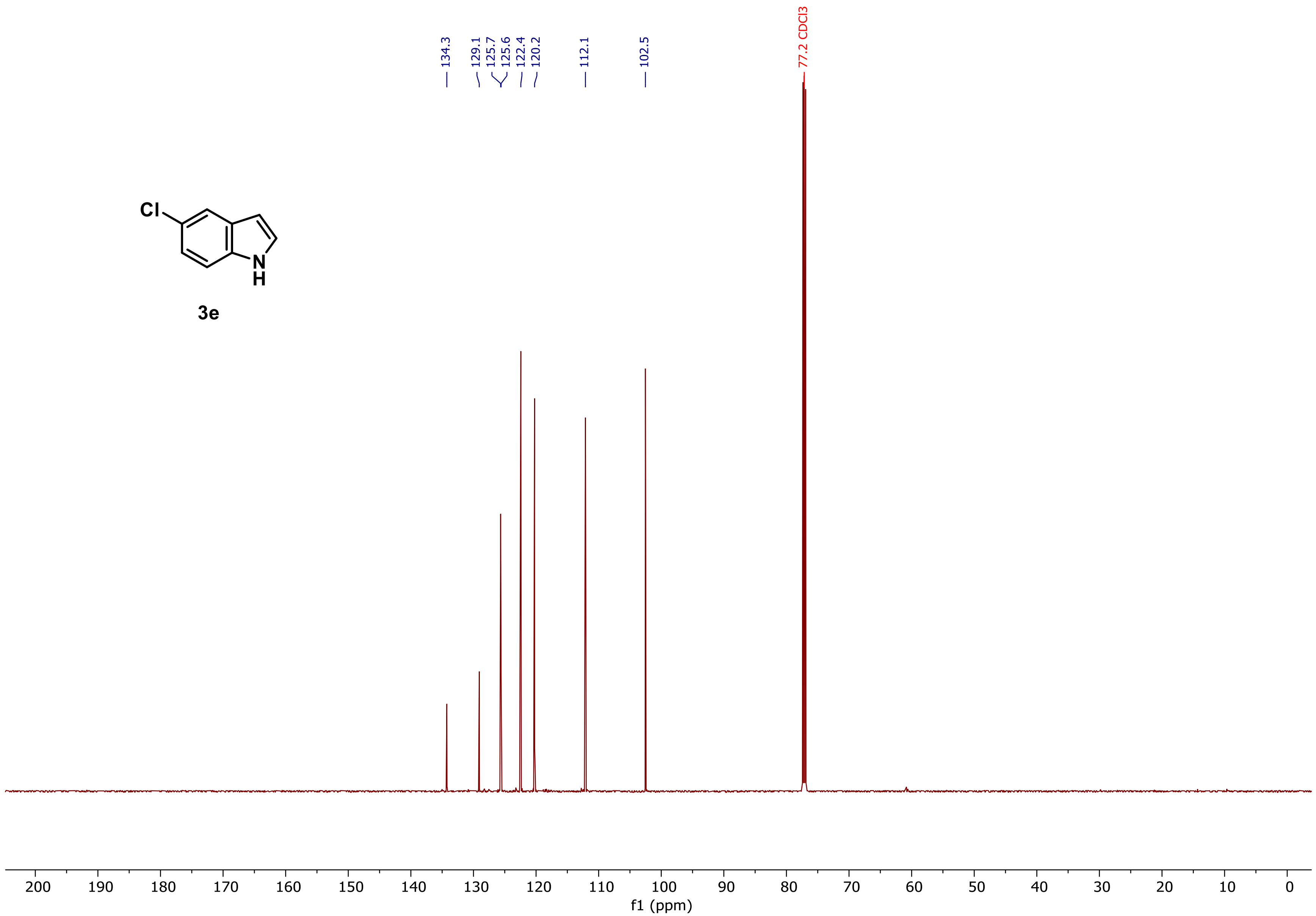

Figure S12: ^{13}C NMR spectrum of **3e**.

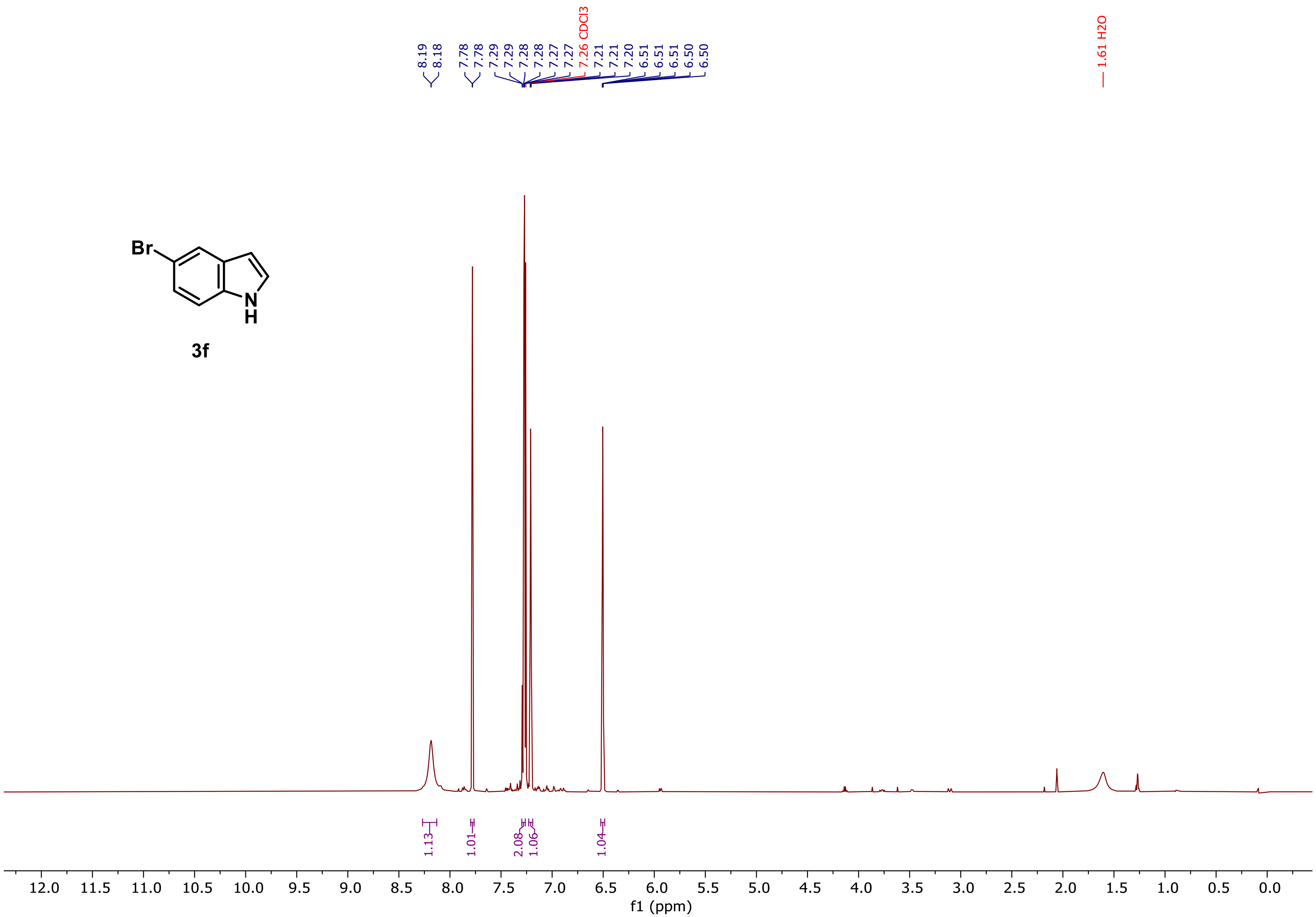

Figure S13: ^{1}H NMR spectrum of **3f**.

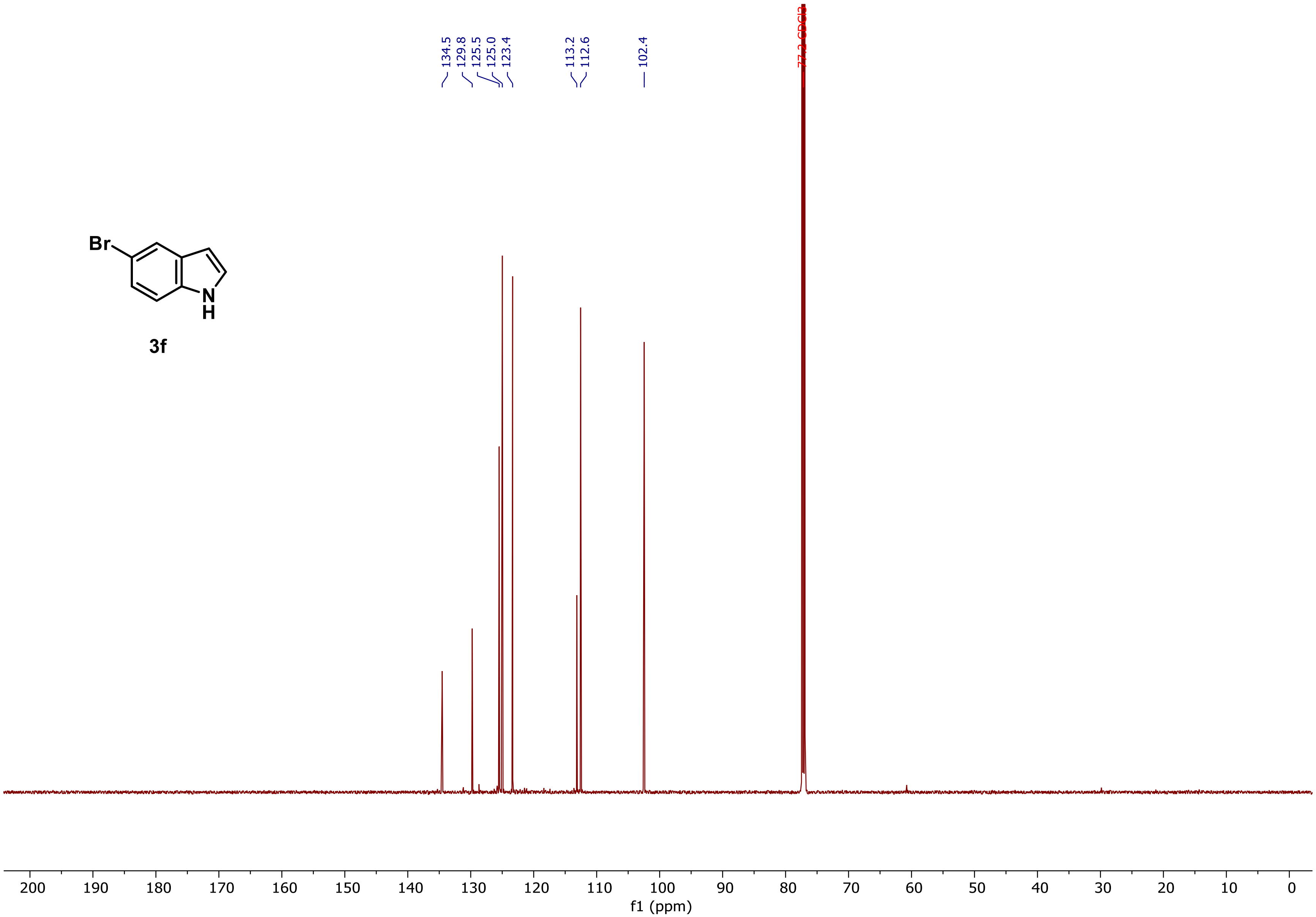

3f

Figure S14: ^{13}C NMR spectrum of **3f**.

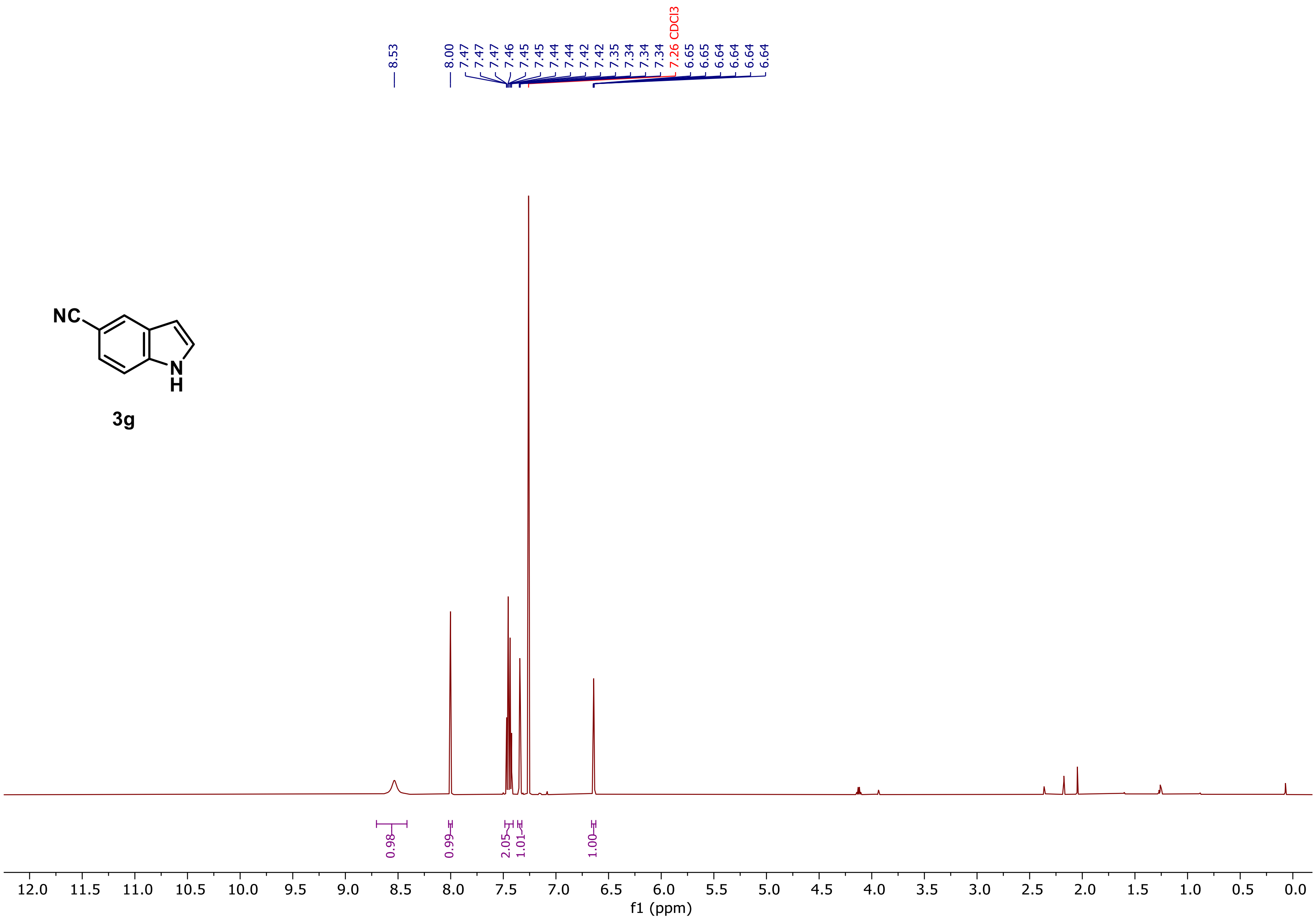

Figure S15: ^{1}H NMR spectrum of **3g**.

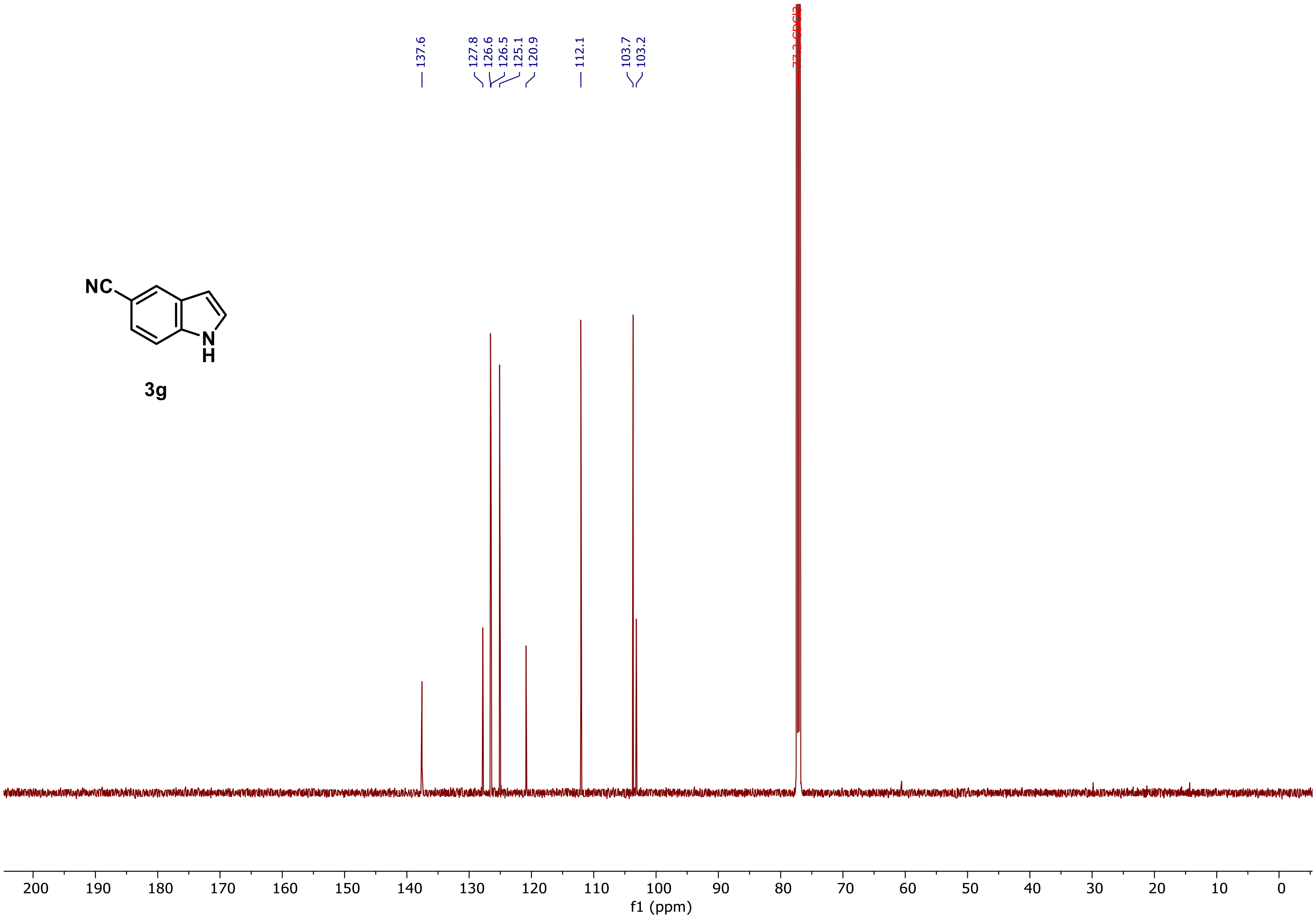

3g

Figure S16: ^{13}C NMR spectrum of **3g**.

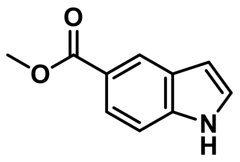

3h

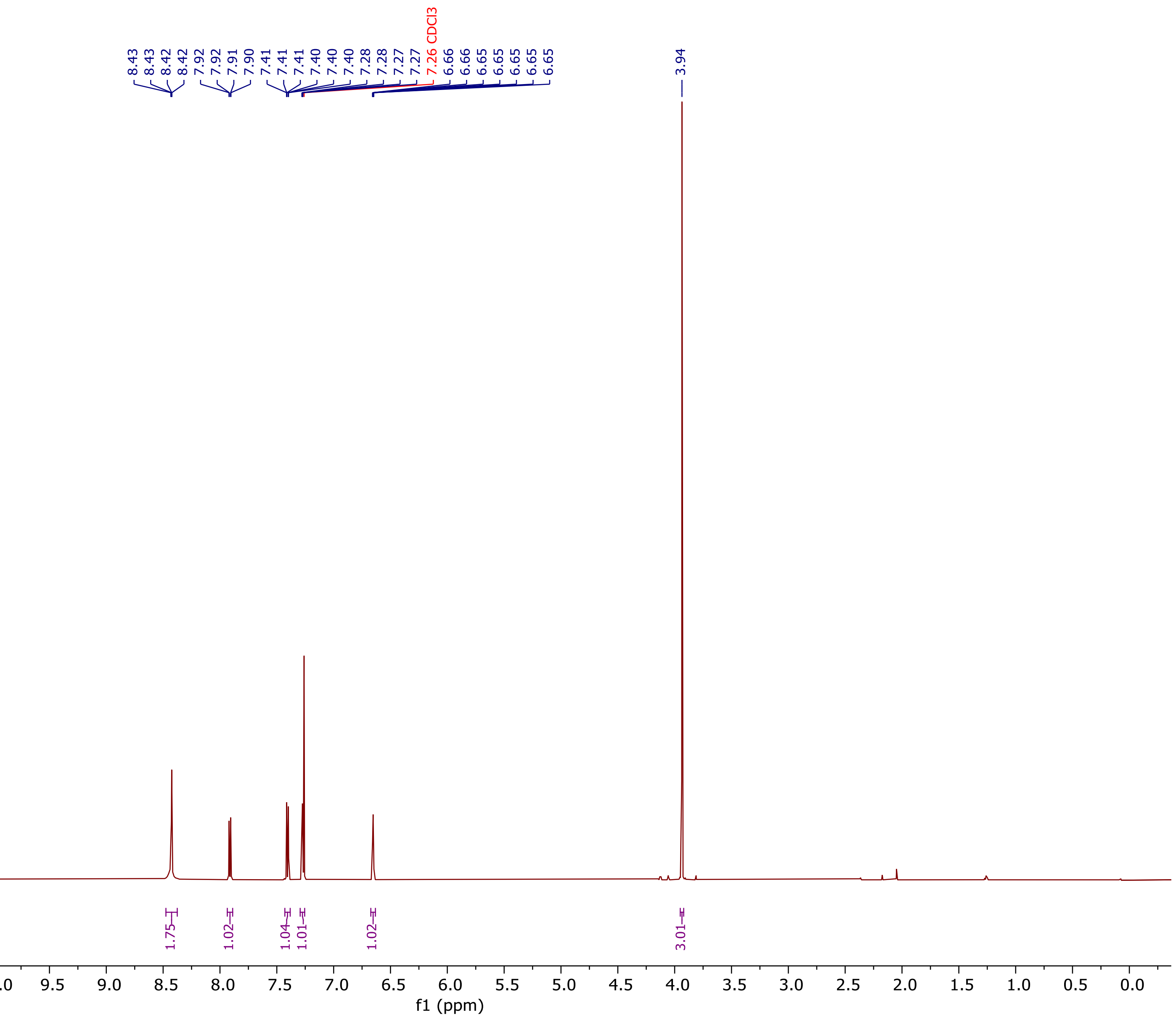

Figure S17: ^{1}H NMR spectrum of **3h**.

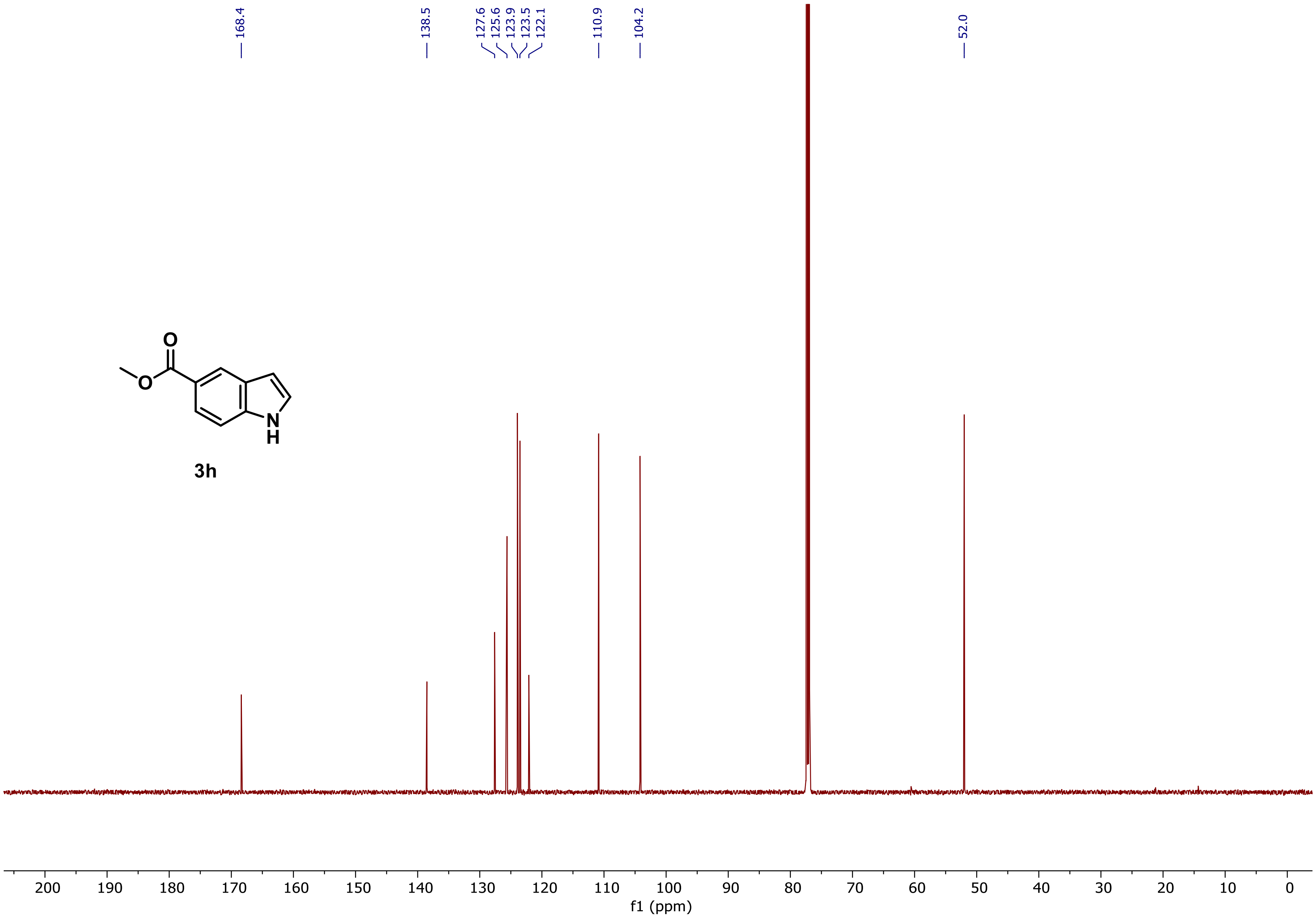

3h

Figure S18: ^{13}C NMR spectrum of **3h**.

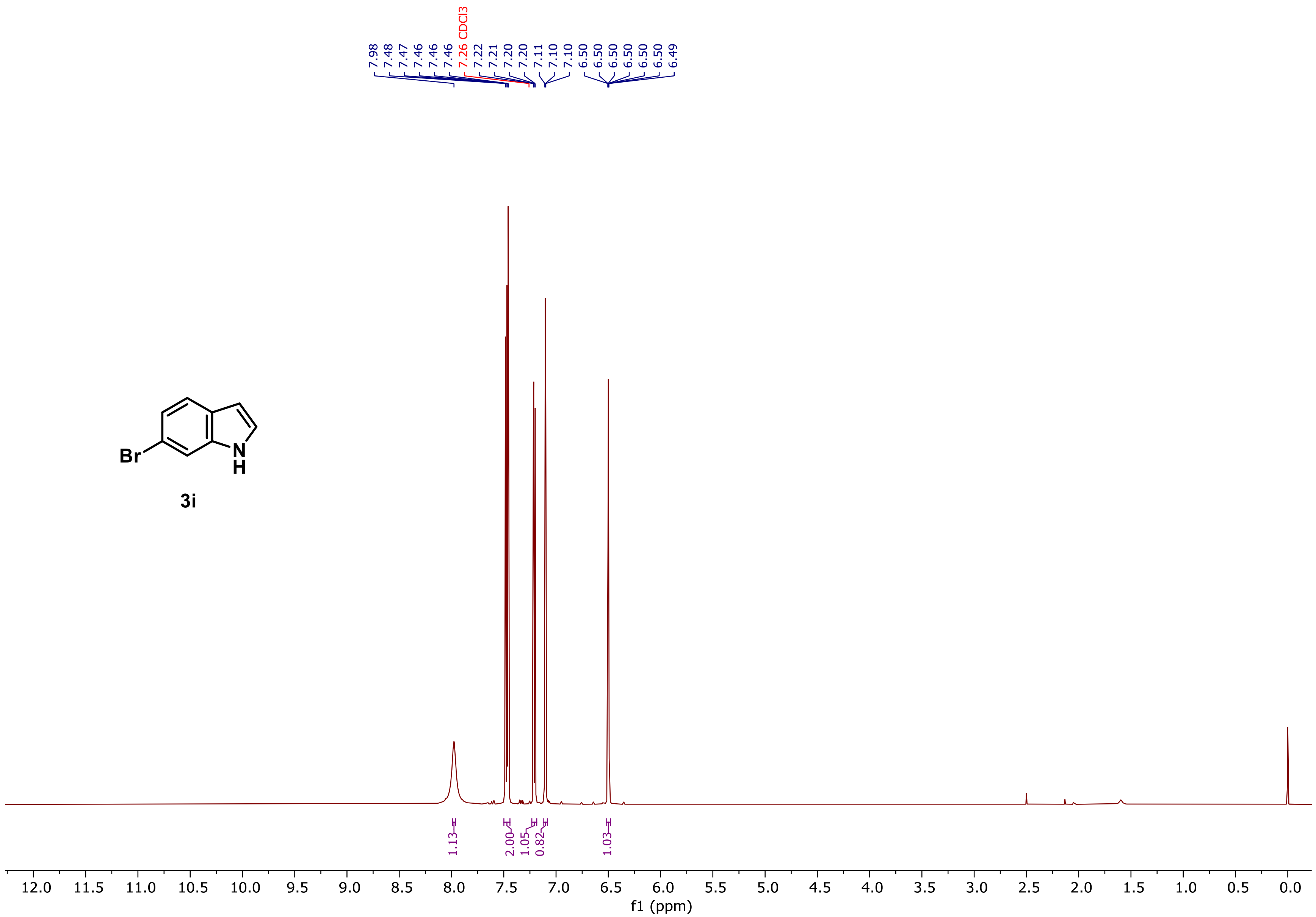

Figure S19: ^{1}H NMR spectrum of **3i**.

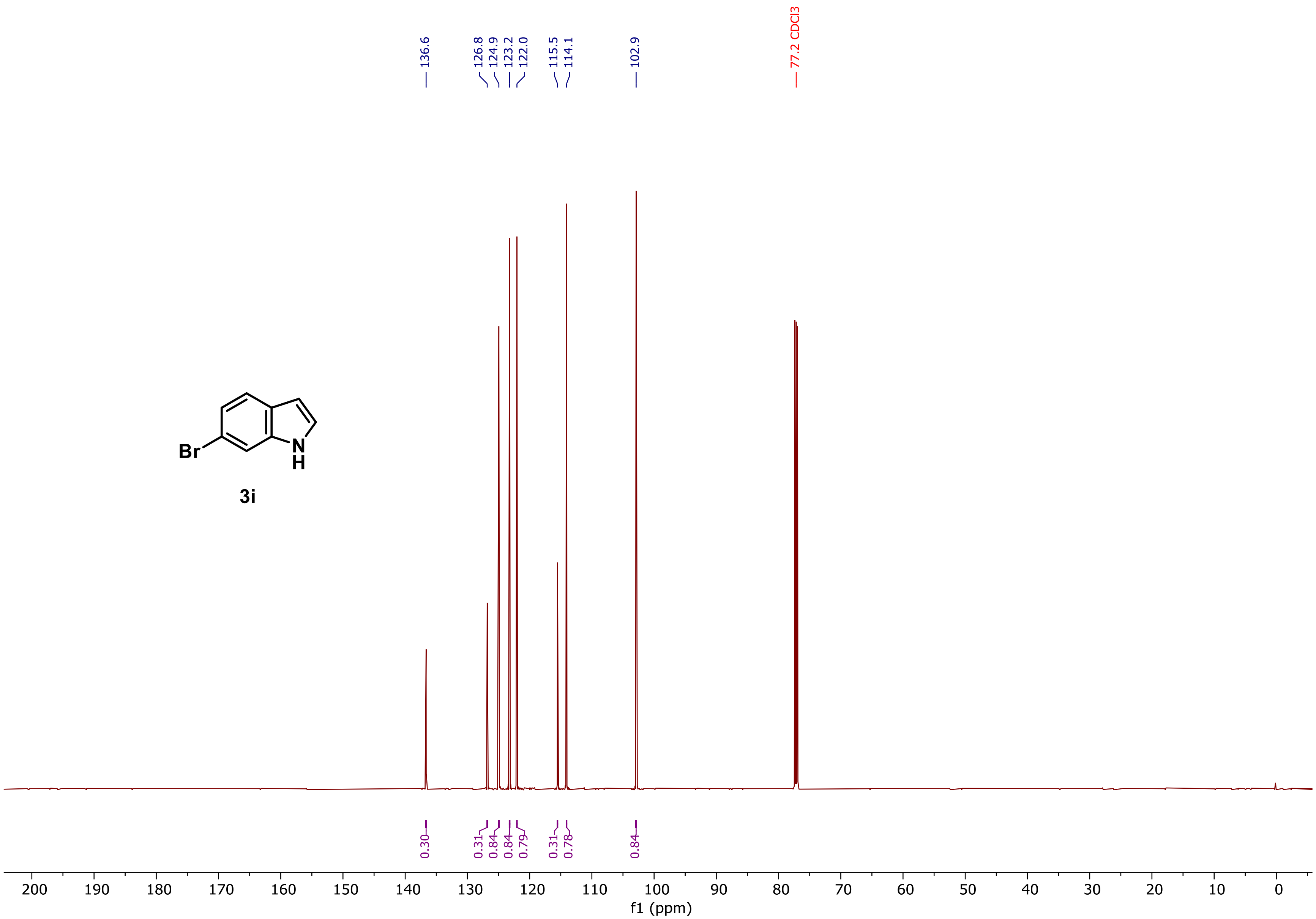

Figure S20: ^{13}C NMR spectrum of **3i**.

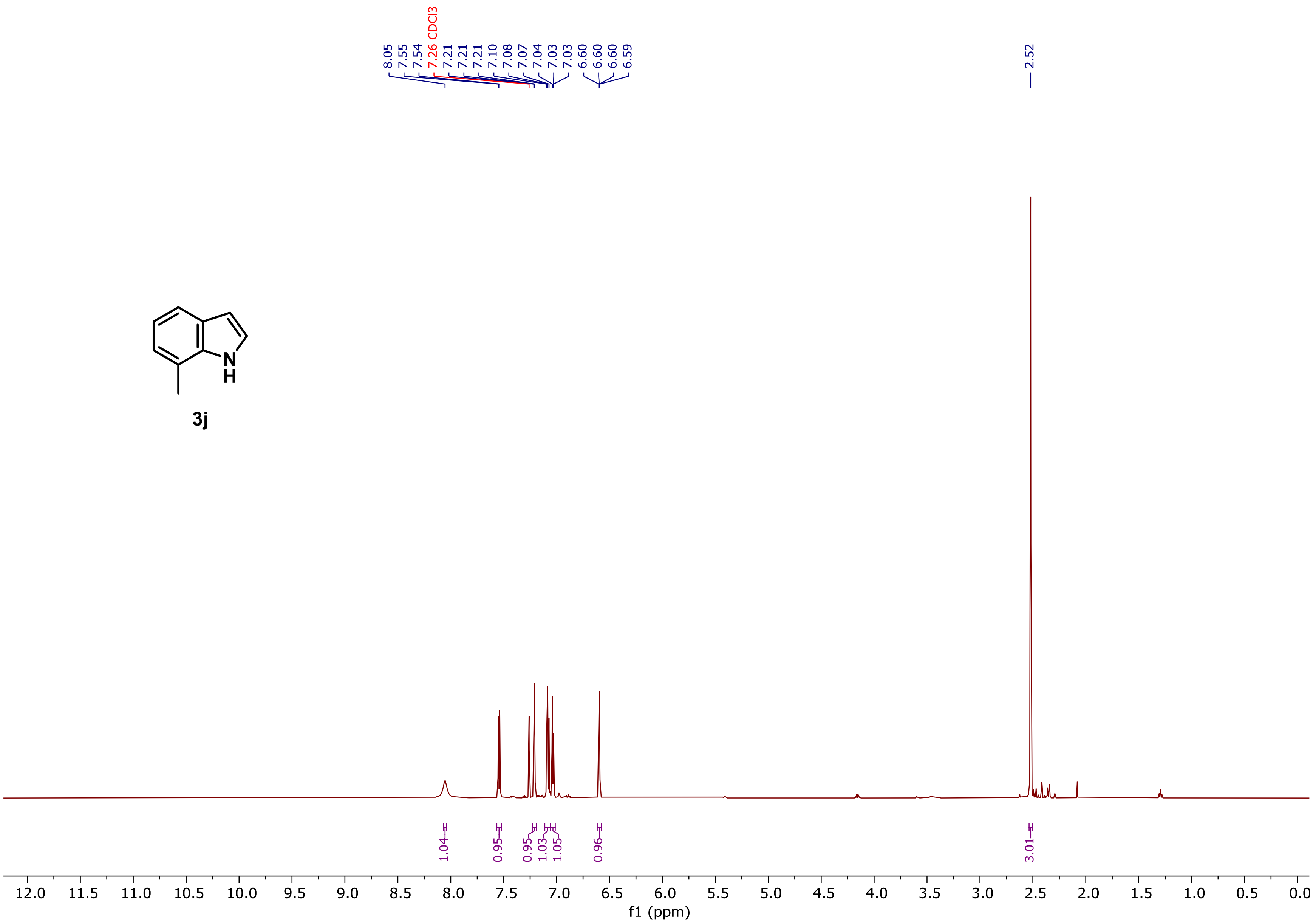

Figure S21: ^{1}H NMR spectrum of **3j**.

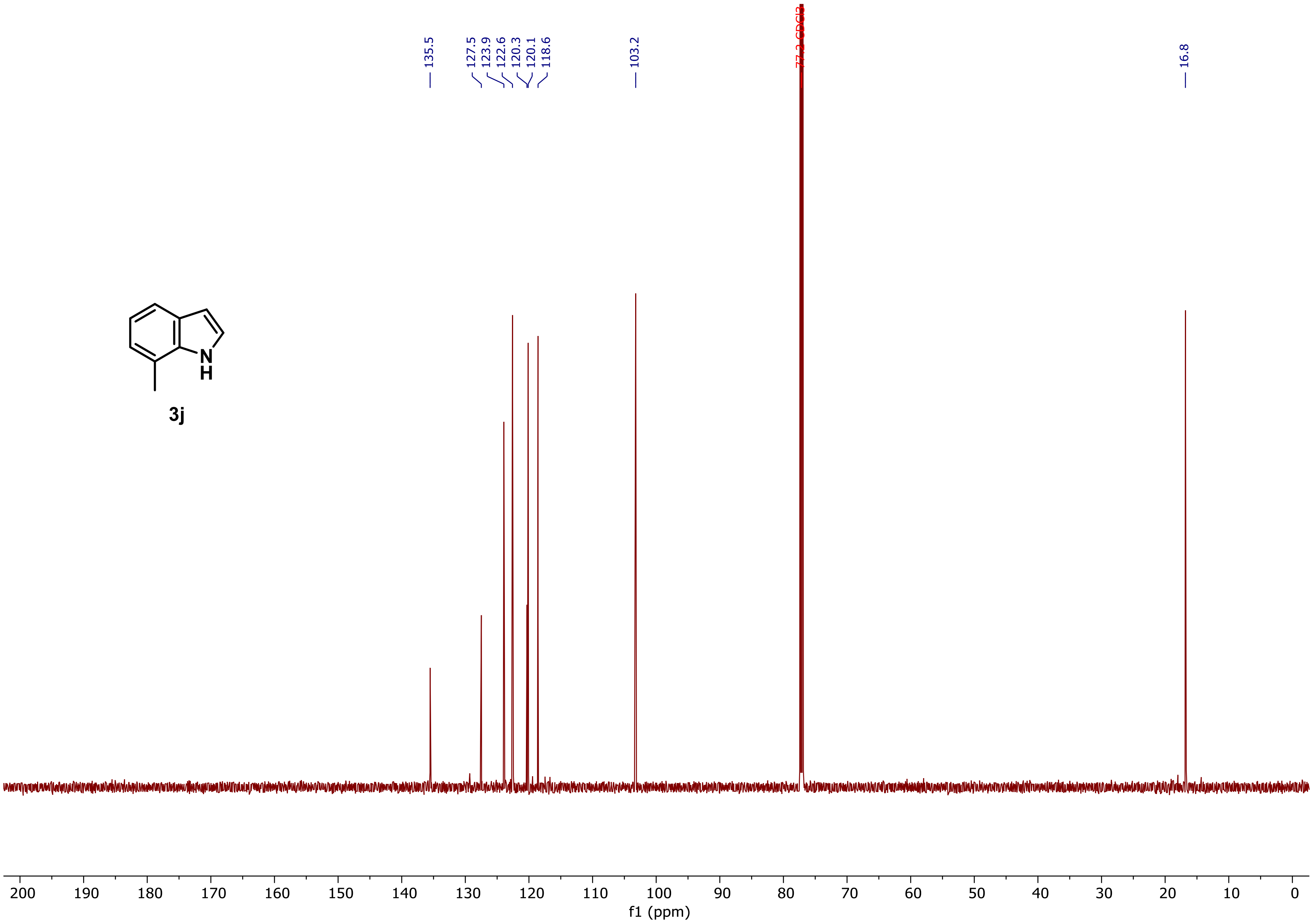

Figure S22: ^{13}C NMR spectrum of **3j**.

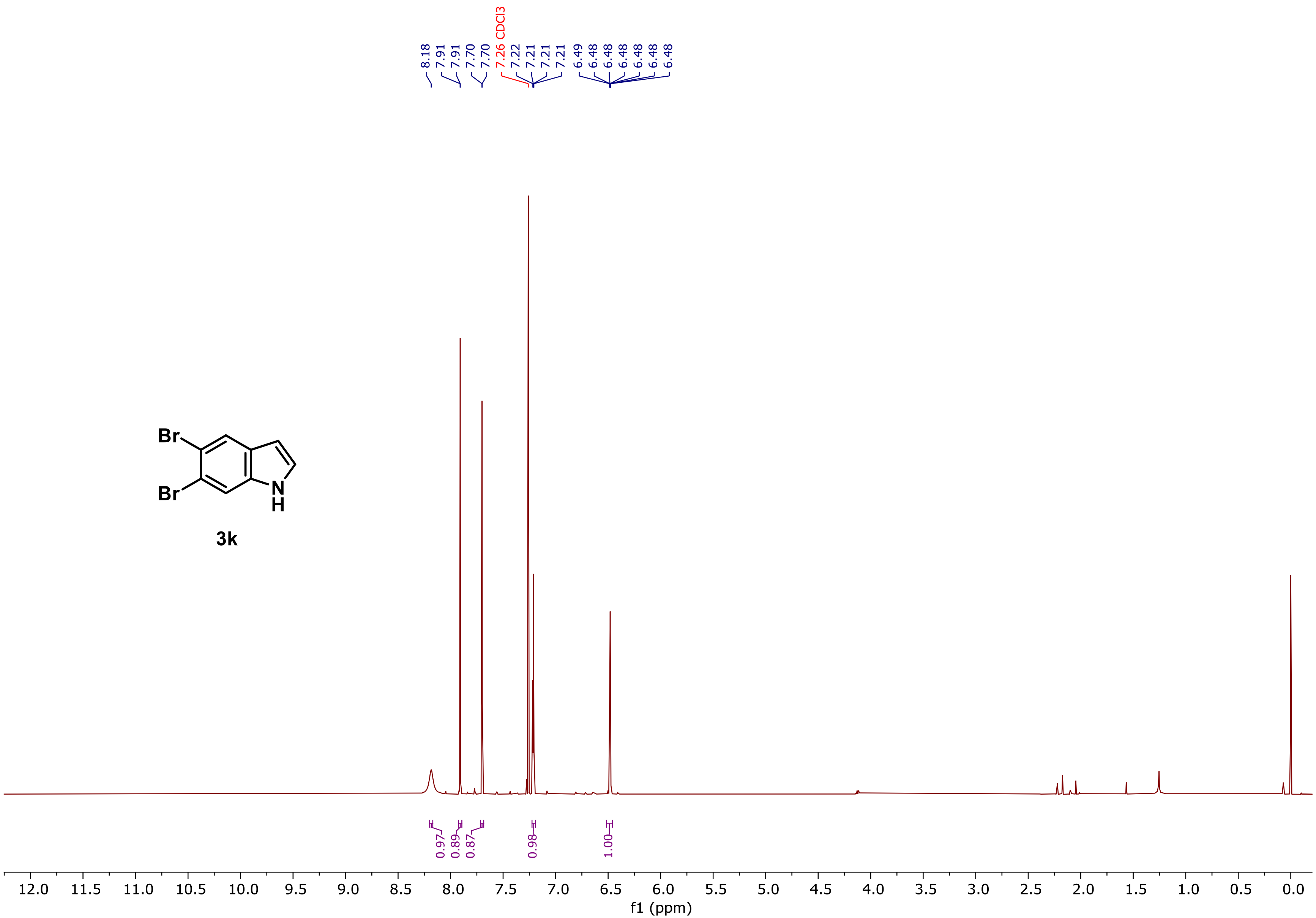

Figure S23: ^{1}H NMR spectrum of **3k**.

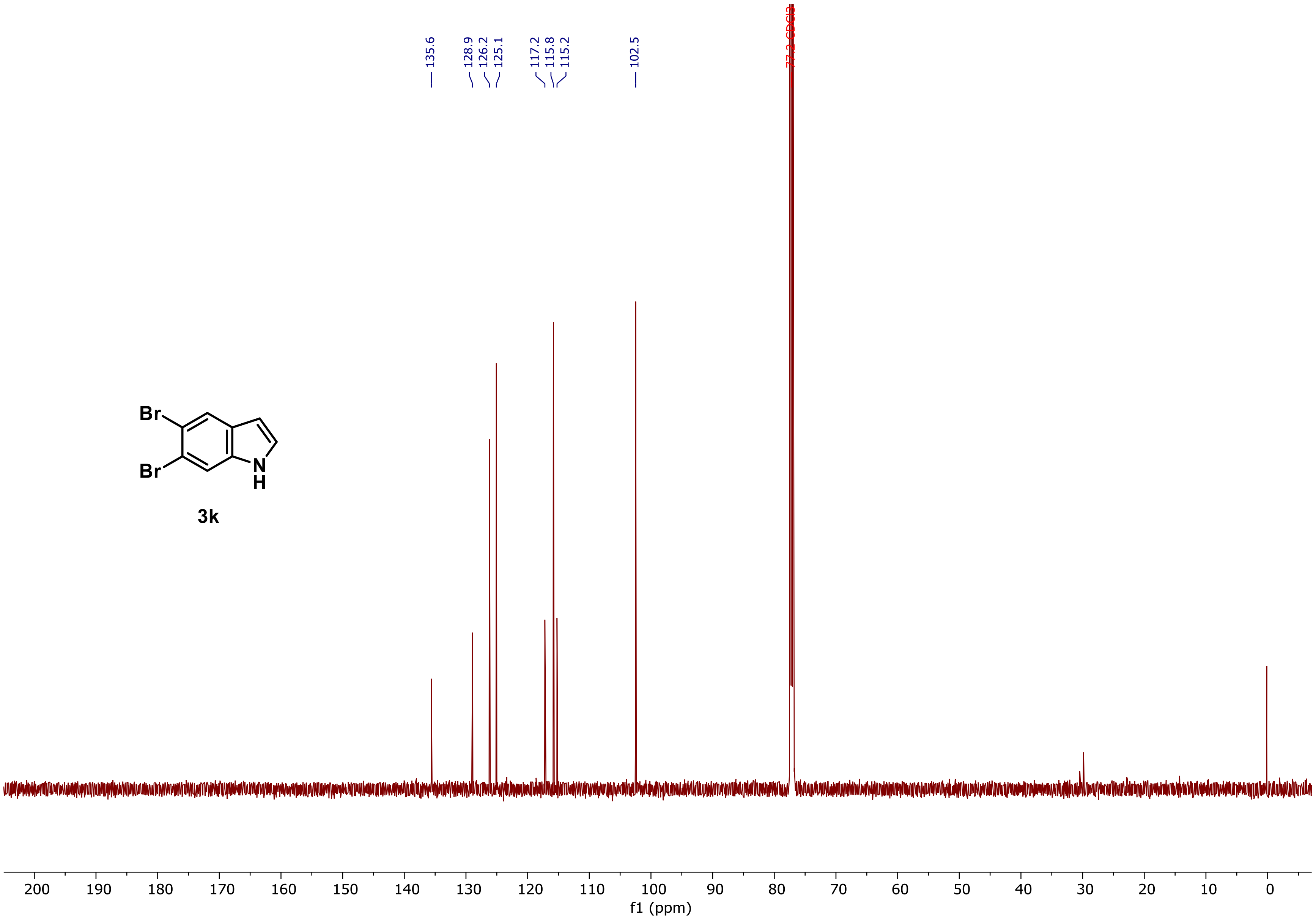

Figure S24: ^{13}C NMR spectrum of **3k**.

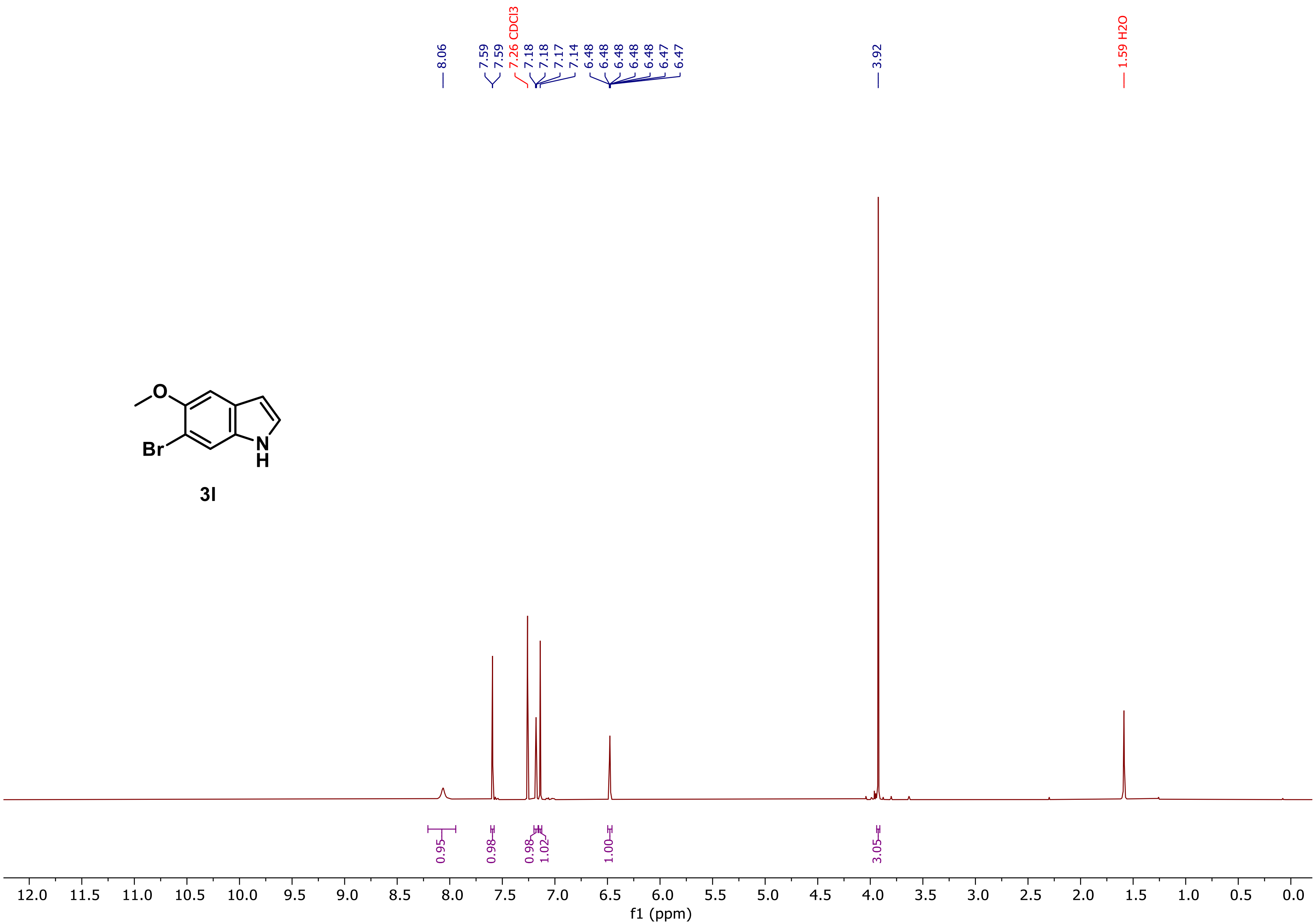

Figure S25: ^{1}H NMR spectrum of **3l**.

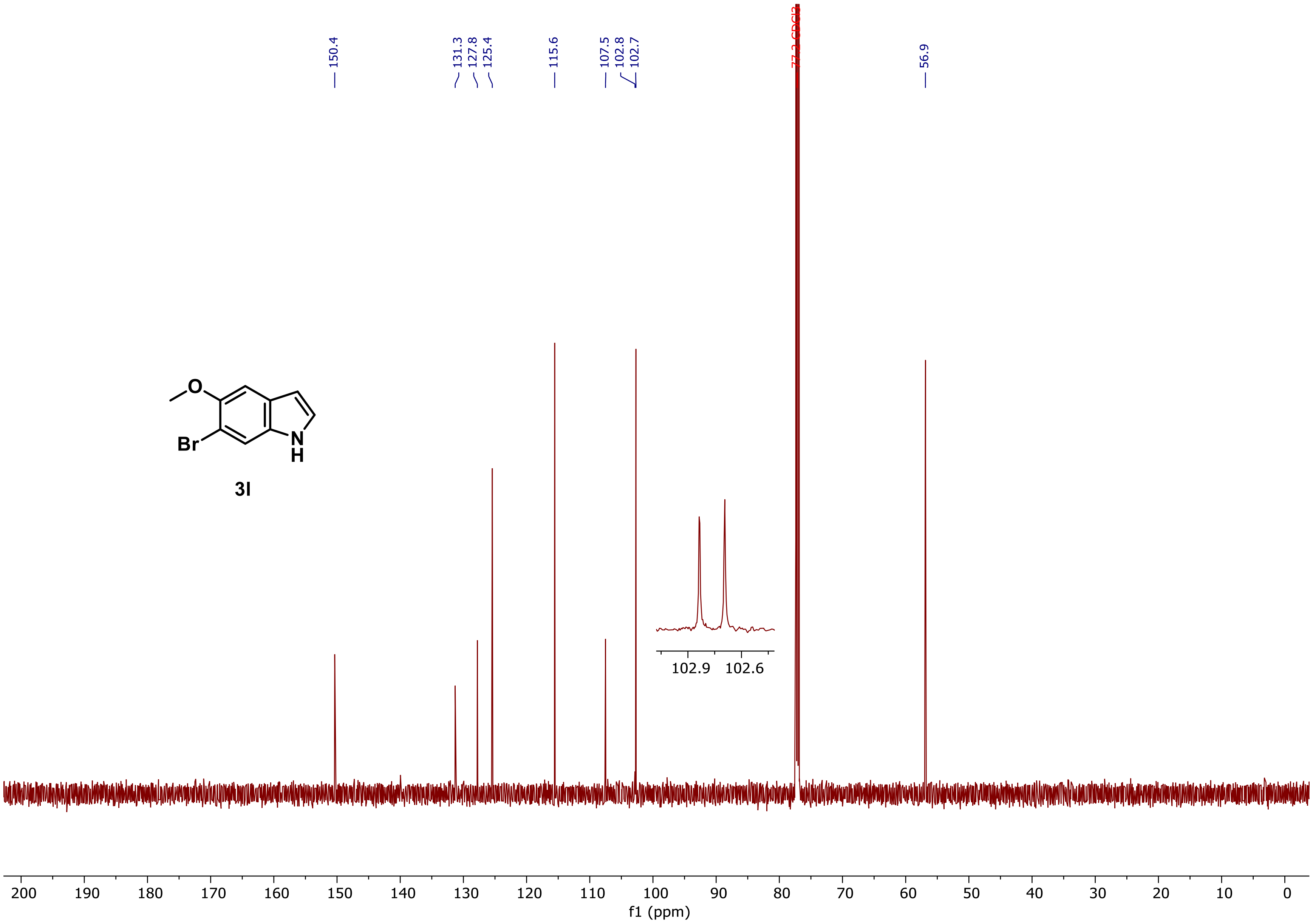

Figure S26: ^{13}C NMR spectrum of **3l**.

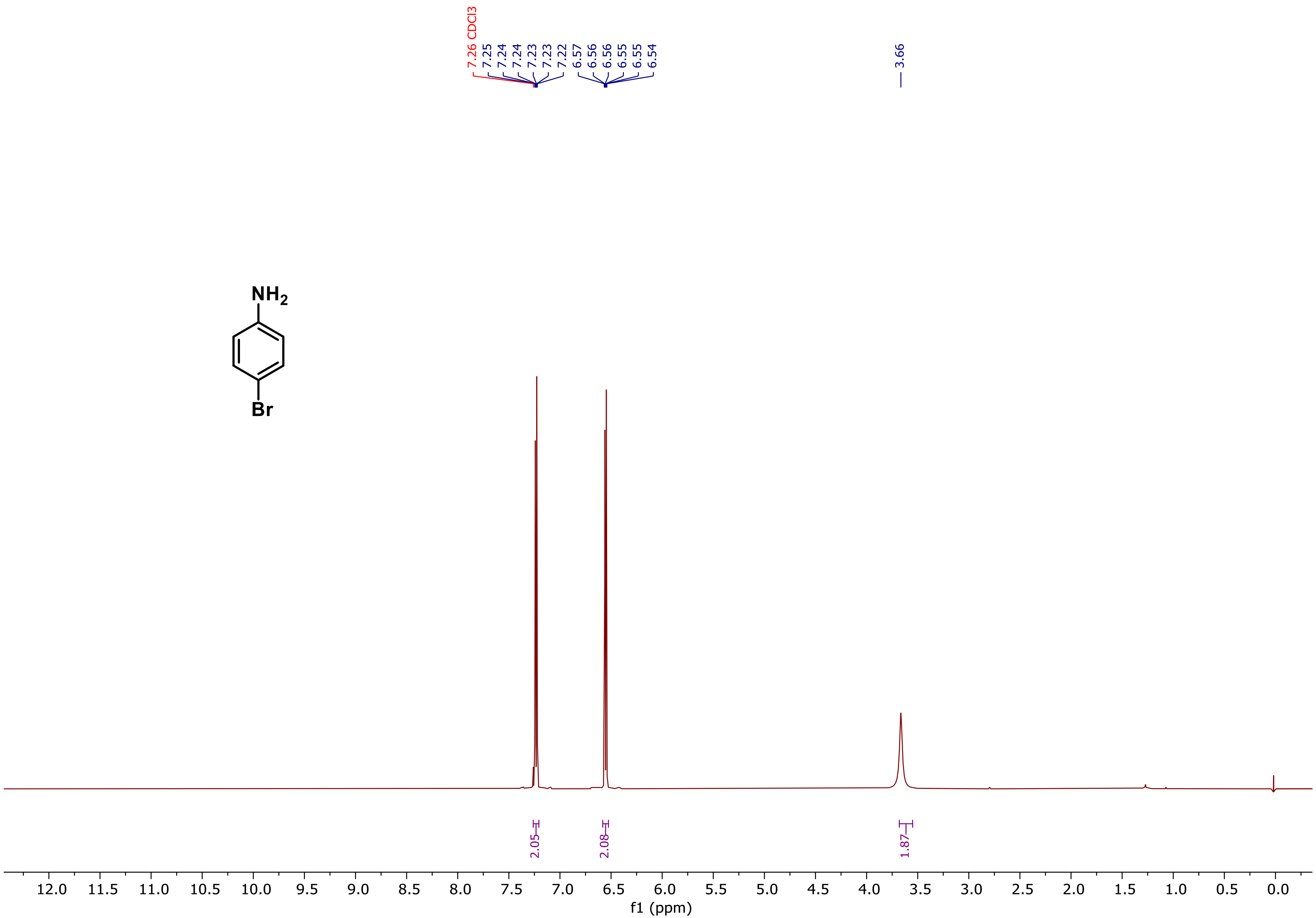

Figure S27: ^{1}H NMR spectrum of 4-bromoaniline.

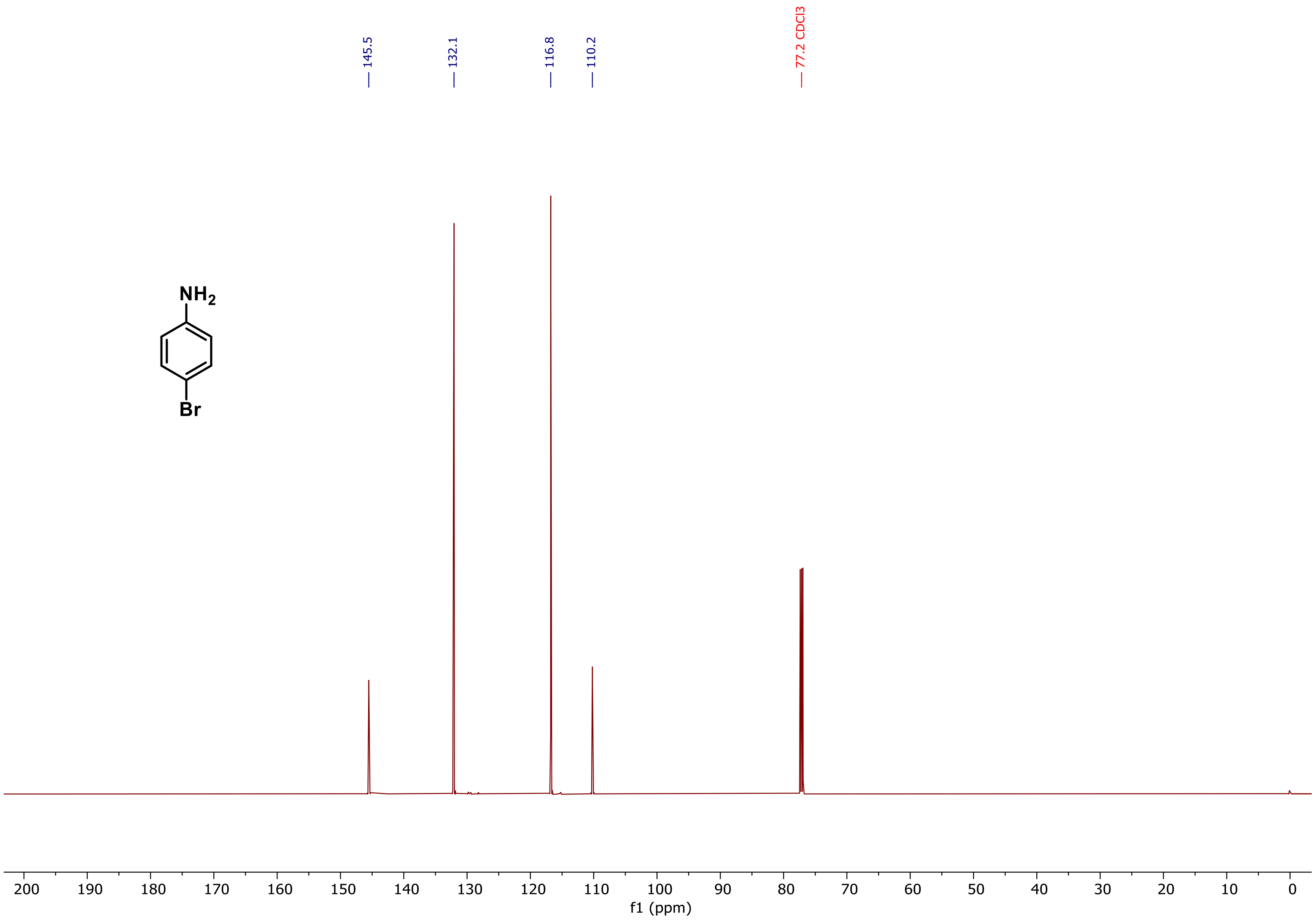

Figure S28: ^{13}C NMR spectrum of 4-bromoaniline.

References

(1) Lankalapalli, R. S.; Ouro, A.; Arana, L.; Gómez-Muñoz, A.; Bittman, R. *J. Org. Chem.* **2009**, *74* (22), 8844–8847.

(2) Chen, J.; Zhang, Z.; Liu, S.; Yang, C.; Xia, C. *RSC Adv.* **2014**, *4* (9), 4672–4675.

(3) Suárez-Castillo, O. R.; Beiza-Granados, L.; Meléndez-Rodríguez, M.; Álvarez-Hernández, A.; Morales-Ríos, M. S.; Joseph-Nathan, P. *J. Nat. Prod.* **2006**, *69* (11), 1596–1600.

(4) Knepper, K.; Bräse, S. *Org. Lett.* **2003**, *5* (16), 2829–2832.

(5) Murcia, C.; Coello, L.; Fernández, R.; Martín, M.; Reyes, F.; Francesch, A.; Munt, S.; Cuevas, C. *Mar. Drugs* **2014**, *12* (2), 1116–1130.

(6) Fukuyama, Y.; Iwatsuki, C.; Kodama, M.; Ochi, M.; Kataoka, K.; Shibata, K. *Tetrahedron* **1998**, *54* (34), 10007–10016.

(7) Jang, M.; Lim, T.; Park, B. Y.; Han, M. S. *J. Org. Chem.* **2022**, *87* (2), 910–919.
